# Supplementary material for: Patient and physician expectations regarding disease and treatment of advanced HCC: The prospective PERCEPTION1 study
Source: JHEP Rep. 2024 Aug 22;6(11):101192. doi: 10.1016/j.jhepr.2024.101192 (PMC11686048; doi:10.1016/j.jhepr.2024.101192)
Supplement: Multimedia component 1 [file mmc1.pdf]

# Patient and physician expectations regarding disease and treatment of advanced HCC: The prospective PERCEPTION1 study

Jean-Charles Nault, Nanthara Sritharan, Gontran Verset, Ivan Borbath, Marie Lequoy, Manon Allaire, Hélène Regnault, Isabelle Colle, Hans Orlent, Isabelle Sinapi, Christophe Moreno, Edouard Larrey, Sabrina Sidali, Clémence Hollande, Giuliana Amaddeo, Stanislas Pol, Pierre Nahon, Nathalie Ganne-Carrié, Vincent Levy, Coralie Bloch-Queyrat, *Paris Liver Cancer Group*, Eric Trepo, Mohammed Bouattour

Table of contents

Fig. S1……………………………………………………………………………..2

Table S1………………………………………………………………………………3

Table S2………………………………………………………………………………4

Table S3………………………………………………………………………………7

Table S4………………………………………………………………………………9

Table S5……………………………………………………………………………10

Table S6……………………………………………………………………………11

Table S7……………………………………………………………………………12

**Fig. S1. Flowchart of the study.**

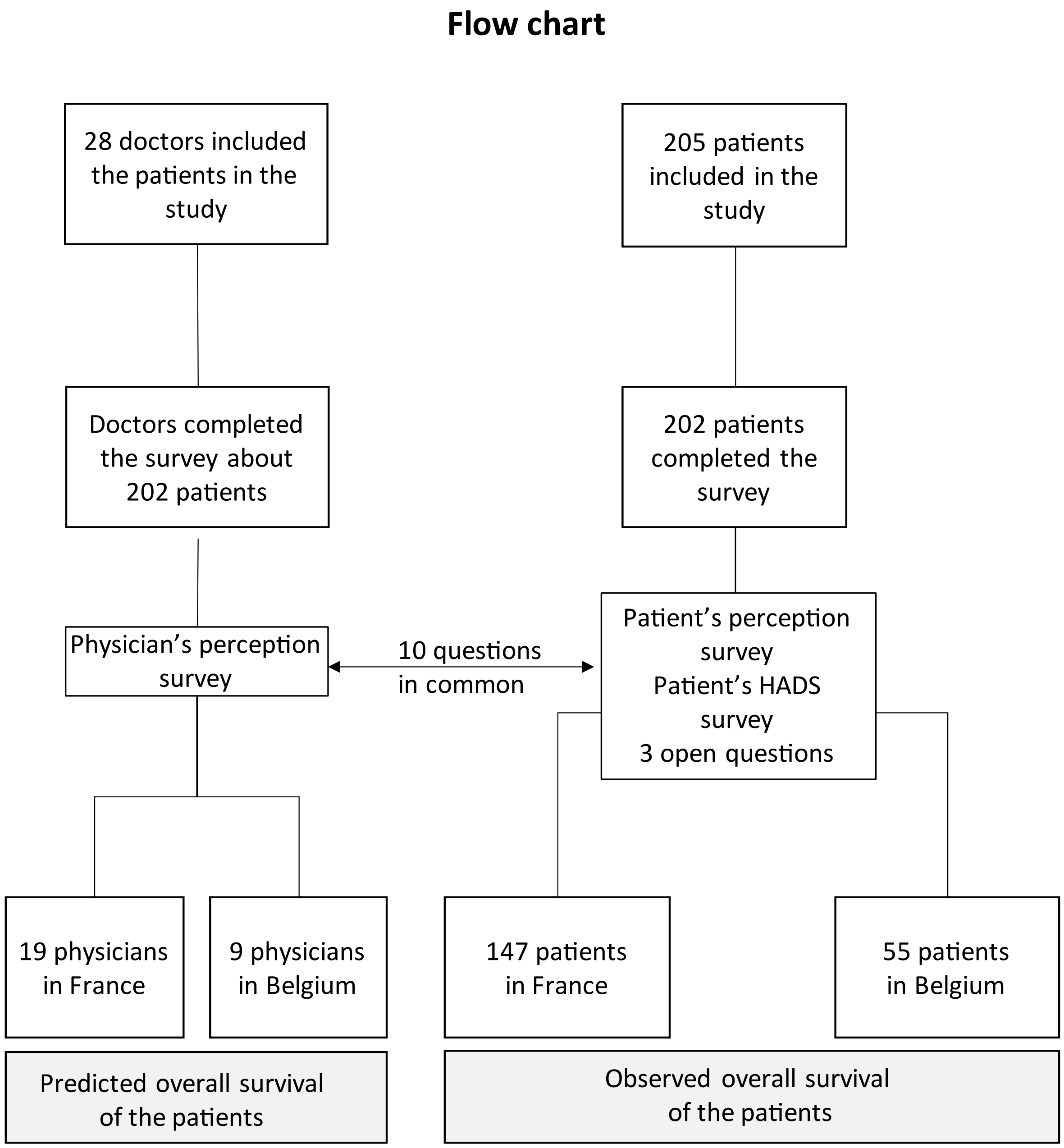

**Table S1: Description of the physicians including patients in the PERCEPTION1 study**

| | Total<br>N= 28 physicians | Belgium<br>N= 9 physicians | France<br>N= 19 physicians |
|---|---|---|---|
| | | | |
| **Gender*** | | | |
| Male | 13 (46.4%) | 7 (77.8%) | 6 (31.6%) |
| Female | 15 (53.6%) | 2 (22.2%) | 13 (68.4%) |
| | | | |
| **Age of the physician (year olds)** $ | 43.0 [38.0;50.2] | 51.0 [43.0;53.0] | 41.0 [37.5;48.0] |
| | | | |
| **Medical Specialty*** | | | |
| Hepato-gastroenterology | 26 (92.9%) | 7 (77.8%) | 19 (100.0%) |
| Medical oncology | 2 (7.1%) | 2 (22.2%) | 0 (0%) |
| | | | |
| **Date of medical graduation*** | | | |
| Less than 5 years | 7 (25.0%) | 0 (0%) | 7 (36.8%) |
| Between 5 and 10 years | 6 (21.4%) | 2 (22.2%) | 4 (21.1%) |
| Between 10 and 20 years | 8 (28.6%) | 3 (33.3%) | 5 (26.3%) |
| Between 20 and 30 years | 6 (21.4%) | 4 (44.4%) | 2 (10.5%) |
| More than 30 years | 1 (3.6%) | 0 (0%) | 1 (5.3%) |
| | | | |
| **Numbers of patients treated for HCC each years*** | | | |
| Less than 5 | 0 (0%) | 0 (0%) | 0 (0%) |
| Between 5 and 20 | 9 (32.1%) | 7 (77.8%) | 2 (10.5%) |
| Between 20 and 50 | 5 (17.9%) | 0 (0.0%) | 5 (26.3%) |
| Between 50 and 100 | 6 (21.4%) | 1 (11.1%) | 5 (26.3%) |
| More than 100 | 8 (28.6%) | 1 (11.1%) | 7 (36.8%) |

*numbers (percentage) $ median (interquartile range)

HCC = hepatocellular carcinoma

## Table S2: Results of the physician's survey

| | Total N= 205 patients | Belgium N= 55 patients | France N= 150 patients | p | Available data |
|---|---|---|---|---|---|
| **Survey completed *** | 202 (98.5%) | 54 (98.2%) | 148 (98.7%) | - | 205 |
| **Consultation for the announcement of cancer's treatment** | | | | | |
| **1. Length of the consultation - minutes**[$] | 30.0 [25.0;35.0] | 30.0 [20.0;35.0] | 30.0 [25.0;36.2] | 0.06 | 197 |
| **2. Patient accompanied during the consultation*** | | | | | |
| No; alone during the consultation | 98 (48.5%) | 21 (38.9%) | 77 (52.0%) | 0.19 | 202 |
| Yes, by their spouse or another family member | 102 (50.5%) | 33 (61.1%) | 69 (46.6%) | | |
| Yes, by a friend | 2 (1.0%) | 0 (0%) | 2 (1.4%) | | |
| **3. Presence of a dedicated nurse during the consultation*** | | | | | |
| During the consultation | 46 (22.8%) | 16 (29.6%) | 30 (20.3%) | 0.001 | 202 |
| After the consultation only | 95 (47.0%) | 14 (25.9%) | 81 (54.7%) | | |
| No consultation nurse | 61 (30.2%) | 24 (44.4%) | 37 (25.0%) | | |
| **4. Do you feel you had enough time to discuss your patient's illness? ***[%] | | | | | |
| Yes, completely | 91 (45.0%) | 28 (51.9%) | 63 (42.6%) | 0.26 | 202 |
| Yes, somewhat | 100 (49.5%) | 22 (40.7%) | 78 (52.7%) | | |
| No, not really | 11 (5.4%) | 4 (7.4%) | 7 (4.7%) | | |
| No, not at all | 0 (0%) | 0 (0%) | 0 (0%) | | |
| **5. Do you think the patient understood what their treatment will involve based on the explanations you provided and the terms used?***[%] | | | | | |
| Yes, completely | 56 (27.7%) | 18 (33.3%) | 38 (25.7%) | 0.14 | 202 |
| Yes, somewhat | 129 (63.9%) | 29 (53.7%) | 100 (67.6%) | | |
| No, not really | 17 (8.4%) | 7 (13.0%) | 10 (6.8%) | | |
| No, not at all | 0 (0%) | 0 (0%) | 0 (0%) | | |
| **6. Do you think you were attentive enough to all the requests the patient wanted to express?***[%] | | | | | |
| Yes, completely | 64 (31.7%) | 27 (50.0%) | 37 (25.0%) | 0.002 | 202 |
| Yes, somewhat | 132 (65.3%) | 25 (46.3%) | 107 (72.3%) | | |
| No, not really | 6 (3.0%) | 2 (3.7%) | 4 (2.7%) | | |
| No, not at all | 0 (0%) | 0 (0%) | 0 (0%) | | |
| **7. How did you inform the patient about the possibility of experiencing side effects related to the prescribed treatment?***[%] | | | | | |
| In detail | 81 (40.5%) | 29 (54.7%) | 52 (35.4%) | 0.03 | 200 |
| Partially, briefly | 113 (56.5%) | 24 (45.3%) | 89 (60.5%) | | |
| I did not mention it | 6 (3.0%) | 0 (0%) | 6 (4.1%) | | |
| **8. Did you provide an estimation of life expectancy?***[%] | | | | | |
| Yes, precisely | 13 (6.5%) | 5 (9.4%) | 8 (5.4%) | 0.001 | 201 |
| Yes, vaguely | 87 (43.3%) | 33 (62.3%) | 54 (36.5%) | | |
| No, not at all | 101 (50.2%) | 15 (28.3%) | 86 (58.1%) | | |
| **9. Do you think the patient trusts your judgment and the treatment you have proposed?***[%] | | | | | |
| Yes, completely | 50 (25.0%) | 24 (45.3%) | 26 (17.7%) | <0.001 | 200 |

| | Total N= 205 patients | Belgium N= 55 patients | France N= 150 patients | p | Available data |
|---|---|---|---|---|---|
| Yes, somewhat | 141 (70.5%) | 27 (50.9%) | 114 (77.6%) | | |
| No, not really | 4 (2.0%) | 0 (0%) | 4 (2.7%) | | |
| No, not at all | 1 (0.5%) | 0 (0%) | 1 (0.7%) | | |
| I don't know | 4 (2.0%) | 2 (3.8%) | 2 (1.4%) | | |
| **Understanding of the illness** | | | | | |
| **1. In your opinion, which option best corresponds to your patient's perception of their illness?*** | | | | | |
| Accurate perception of the diagnosis | 74 (36.8%) | 11 (20.8%) | 63 (42.6%) | 0.01 | 201 |
| Partially accurate perception | 122 (60.7%) | 40 (75.5%) | 82 (55.4%) | | |
| Inaccurate perception of the diagnosis | 5 (2.5%) | 2 (3.8%) | 3 (2.0%) | | |
| **2. Do you think your patient has realistic expectations regarding the prescribed treatment?*** | | | | | |
| Yes | 158 (78.6%) | 31 (58.5%) | 127 (85.8%) | <0.001 | 201 |
| **3. Do you think the patient's perception of the progression of their illness and overall condition over the next 12 months is realistic? *** | | | | | |
| Yes | 119 (61.0%) | 27 (50.9%) | 92 (64.8%) | 0.11 | 195 |
| **4. In your opinion, what is the life expectancy of the patient? *%** | | | | | |
| more than 5 years | 0 (0%) | 0 (0%) | 0 (0%) | 0.04 | 201 |
| Between 2 à 5 years | 43 (21.4%) | 16 (30.2%) | 27 (18.2%) | | |
| Between 1 à 2 years | 109 (54.2%) | 30 (56.6%) | 79 (53.4%) | | |
| Less than 1 year | 49 (24.4%) | 7 (13.2%) | 42 (28.4%) | | |
| **5. In your opinion, what is the likelihood that your patient will be alive in 2 years? *** | | | | | |
| 100% | 0 (0%) | 0 (0%) | 0 (0%) | <0.001 | 202 |
| 75 to 100% | 13 (6.4%) | 9 (16.7%) | 4 (2.7%) | | |
| 50 to 75% | 43 (21.3%) | 17 (31.5%) | 26 (17.6%) | | |
| 25 to 50% | 63 (31.2%) | 16 (29.6%) | 47 (31.8%) | | |
| 0 to 50% | 83 (41.1%) | 12 (22.2%) | 71 (48.0%) | | |
| **Predicted survival of your patient in months** $ | 17.0 [12.0;21.5] | 18.0 [12.0;23.5] | 16.0 [10.0;20.0] | 0.12 | 198 |
| **7. In your opinion, what is the probability that the treatment you prescribed will control (at least temporarily) the disease?* %** | | | | | |
| 0% | 1 (0.5%) | 0 (0%) | 1 (0.7%) | <0.001 | 202 |
| Between 0 and 25% | 64 (31.7%) | 4 (7.4%) | 60 (40.5%) | | |
| Between 25 and 50% | 64 (31.7%) | 17 (31.5%) | 47 (31.8%) | | |
| Between 50 and 75% | 66 (32.7%) | 29 (53.7%) | 37 (25.0%) | | |
| Between 75 and 100% | 6 (3.0%) | 3 (5.6%) | 3 (2.0%) | | |
| 100% | 1 (0.5%) | 1 (1.9%) | 0 (0%) | | |
| **8. What is the percentage chance that the treatment you prescribed will cause disabling side effects (such as diarrhea, vomiting, significant fatigue, pain...)? *%** | | | | | |
| 0% | 2 (1.0%) | 0 (0%) | 2 (1.4%) | 0.31 | 202 |
| Between 0 and 25% | 111 (55.0%) | 27 (50.0%) | 84 (56.8%) | | |
| Between 25 and 50% | 73 (36.1%) | 20 (37.0%) | 53 (35.8%) | | |
| Between 50 and 75% | 15 (7.4%) | 6 (11.1%) | 9 (6.1%) | | |

| | Total<br>N= 205 patients | Belgium<br>N= 55 patients | France<br>N= 150 patients | p | Available data |
|---|---|---|---|---|---|
| Between 75 and 100% | 1 (0.5%) | 1 (1.9%) | 0 (0%) | | |
| 100% | 0 (0%) | 0 (0%) | 0 (0%) | | |
| **9. Do you believe that for your patient, maintaining quality of life is more important than prolonging life? *%** | | | | | |
| Yes, absolutely | 55 (27.2%) | 10 (18.5%) | 45 (30.4%) | | |
| Yes, to some extent | 90 (44.6%) | 26 (48.1%) | 64 (43.2%) | | |
| No, probably not | 36 (17.8%) | 4 (7.4%) | 32 (21.6%) | <0.001 | 202 |
| No, not at all | 7 (3.5%) | 2 (3.7%) | 5 (3.4%) | | |
| I don't know | 14 (6.9%) | 12 (22.2%) | 2 (1.4%) | | |

*numbers (percentage) $ median (interquartile range)

% These 10 questions are used to assess the concordance between the patients and the physicians

## Table S3: Results of the Hospital Anxiety and Depression scale (HADS) survey

| | Total N= 200 patients | Belgium N= 50 patients | France N= 150 patients | Available data |
|---|---|---|---|---|
| **HADS survey completed*** | 202 (98.5%) | 55 (100.0%) | 147 (98.0%) | - |
| **1. I feel tense or 'wound up*** | | | | |
| Most of the time | 19 (9.5%) | 5 (9.4%) | 14 (9.5%) | 200 |
| A lot of the time | 27 (13.5%) | 11 (20.8%) | 16 (10.9%) | |
| Occasionally | 106 (53.0%) | 19 (35.8%) | 87 (59.2%) | |
| Not at all | 48 (24.0%) | 18 (34.0%) | 30 (20.4%) | |
| **2. I still enjoy the things I used to enjoy*** | | | | |
| Definitely as much | 97 (48.5%) | 28 (52.8%) | 69 (46.9%) | 200 |
| Not quite so much | 76 (38.0%) | 22 (41.5%) | 54 (36.7%) | |
| Only a little | 18 (9.0%) | 2 (3.8%) | 16 (10.9%) | |
| Hardly at all | 9 (4.5%) | 1 (1.9%) | 8 (5.4%) | |
| **3. I get a sort of frightened feeling as if something awful is about to happen*** | | | | |
| Very definitely & quite badly | 37 (18.7%) | 9 (17.3%) | 28 (19.2%) | 198 |
| Yes, but not too badly | 36 (18.2%) | 10 (19.2%) | 26 (17.8%) | |
| A little, but it doesn't worry me | 68 (34.3%) | 16 (30.8%) | 52 (35.6%) | |
| Not at all | 57 (28.8%) | 17 (32.7%) | 40 (27.4%) | |
| **4. I can laugh and see the funny side of things*** | | | | |
| As much as always | 108 (54.5%) | 30 (56.6%) | 78 (53.8%) | 198 |
| Not quite so much now | 58 (29.3%) | 16 (30.2%) | 42 (29.0%) | |
| Definitely not so much now | 29 (14.6%) | 6 (11.3%) | 23 (15.9%) | |
| Not at all | 3 (1.5%) | 1 (1.9%) | 2 (1.4%) | |
| **5. Worrying thoughts go through my mind*** | | | | |
| A great deal of the time | 30 (15.5%) | 14 (28.0%) | 16 (11.1%) | 194 |
| A lot of the time | 43 (22.2%) | 7 (14.0%) | 36 (25.0%) | |
| Not too often | 58 (29.9%) | 13 (26.0%) | 45 (31.2%) | |
| Very little | 63 (32.5%) | 16 (32.0%) | 47 (32.6%) | |
| **6. I feel cheerful *** | | | | |
| Never | 1 (0.5%) | 1 (1.9%) | 0 (0.0%) | 198 |
| Not often | 16 (8.1%) | 4 (7.5%) | 12 (8.3%) | |
| Sometimes | 32 (16.2%) | 7 (13.2%) | 25 (17.2%) | |
| Most of the time | 149 (75.3%) | 41 (77.4%) | 108 (74.5%) | |
| **7. I can sit at ease and feel relaxed*** | | | | |
| Definitely | 95 (48.0%) | 27 (50.9%) | 68 (46.9%) | 198 |
| Usually | 75 (37.9%) | 20 (37.7%) | 55 (37.9%) | |
| Not often | 21 (10.6%) | 4 (7.5%) | 17 (11.7%) | |
| Not at all | 7 (3.5%) | 2 (3.8%) | 5 (3.4%) | |
| **8. I feel as if I am slowed down*** | | | | |
| Nearly all the time | 18 (9.1%) | 5 (9.4%) | 13 (9.0%) | 197 |
| Very often | 50 (25.4%) | 11 (20.8%) | 39 (27.1%) | |
| Sometimes | 78 (39.6%) | 17 (32.1%) | 61 (42.4%) | |
| Not at all | 51 (25.9%) | 20 (37.7%) | 31 (21.5%) | |

| | Total N= 200 patients | Belgium N= 50 patients | France N= 150 patients | Available data |
|---|---|---|---|---|
| **9. I get a sort of frightened feeling like 'butterflies' in the stomach*** | | | | |
| Not at all | 91 (46.4%) | 24 (45.3%) | 67 (46.9%) | 196 |
| Occasionally | 88 (44.9%) | 26 (49.1%) | 62 (43.4%) | |
| Quite often | 11 (5.6%) | 2 (3.8%) | 9 (6.3%) | |
| Very often | 6 (3.1%) | 1 (1.9%) | 5 (3.5%) | |
| **10. I have lost interest in my appearance*** | | | | |
| Definitely | 17 (8.6%) | 7 (13.2%) | 10 (6.9%) | 197 |
| I don't take as much care as I should | 28 (14.2%) | 9 (17.0%) | 19 (13.2%) | |
| I may not take as much care | 52 (26.4%) | 7 (13.2%) | 45 (31.2%) | |
| I take just as much care as ever | 100 (50.8%) | 30 (56.6%) | 70 (48.6%) | |
| **11. I feel restless as if I have to be on the move** | | | | |
| Very much indeed | 21 (10.7%) | 6 (11.3%) | 15 (10.5%) | 196 |
| Quite a lot | 33 (16.8%) | 11 (20.8%) | 22 (15.4%) | |
| Not very much | 75 (38.3%) | 15 (28.3%) | 60 (42.0%) | |
| Not at all | 67 (34.2%) | 21 (39.6%) | 46 (32.2%) | |
| **12. I look forward to enjoyment to things*** | | | | |
| As much as I ever did | 76 (39.2%) | 20 (40.0%) | 56 (38.9%) | 194 |
| Rather less than I used to | 79 (40.7%) | 21 (42.0%) | 58 (40.3%) | |
| Definitely less than I used to | 28 (14.4%) | 7 (14.0%) | 21 (14.6%) | |
| Hardly at all | 11 (5.7%) | 2 (4.0%) | 9 (6.2%) | |
| **13. I get sudden feelings of panic*** | | | | |
| Very often indeed | 1 (0.5%) | 1 (1.9%) | 0 (0.0%) | 198 |
| Quite often | 20 (10.1%) | 5 (9.6%) | 15 (10.3%) | |
| Not very often | 62 (31.3%) | 16 (30.8%) | 46 (31.5%) | |
| Not at all | 115 (58.1%) | 30 (57.7%) | 85 (58.2%) | |
| **14. I can enjoy a good book or radio or TV program*** | | | | |
| Often | 137 (69.2%) | 37 (71.2%) | 100 (68.5%) | 198 |
| Sometimes | 44 (22.2%) | 10 (19.2%) | 34 (23.3%) | |
| Not often | 8 (4.0%) | 3 (5.8%) | 5 (3.4%) | |
| Very seldom | 9 (4.5%) | 2 (3.8%) | 7 (4.8%) | |
| **HADS score (anxiety)** $^{\$}$ | 6.0 [4.0;9.0] | 6.0 [3.0;10.0] | 6.0 [4.0;9.0] | 188 |
| **HADS score (depression)** $^{\$}$ | 4.0 [2.0;7.2] | 4.0 [1.2;7.0] | 5.0 [3.0;7.8] | 192 |
| **HADS score (total)** $^{\$}$ | 11.0 [7.0;16.0] | 10.5 [5.2;16.8] | 11.0 [7.0;15.0] | 185 |
| **HADS score by category*** | | | | |
| No symptoms (0 to 7) | 58 (31.4%) | 16 (34.8%) | 42 (30.2%) | 185 |
| Doubtful symptoms (8 to 10) | 30 (16.2%) | 7 (15.2%) | 23 (16.5%) | |
| Certain symptoms (11 to more) | 97 (52.4%) | 23 (50.0%) | 74 (53.2%) | |

*numbers (percentage) $^{\$}$ median (interquartile range), HADS = Hospital Anxiety and Depression Scale

**Table S4: Association between patient's factors and HADS using multivariate linear regression**

| | β ± SE | p |
|---|---|---|
| Country – France (ref : Belgium) | 0.24 ± 1.01 | 0.81 |
| Non tumor fibrosis F2/F3 (ref : F0/F1) | 2.26 ± 1.83 | 0.22 |
| Non tumor fibrosis F4 | 1.70 ± 1.17 | 0.15 |
| Serum AFP level (logarithmic) | 0.34 ± 0.15 | **0.03** |
| Performance status 1/2 (ref : 0) | 1.34 ± 0.94 | 0.15 |
| Unemployed (Job seeker) (ref: active worker) | 2.76 ± 2.18 | 0.21 |
| Unable to work, disabled | 3.63 ± 1.81 | **0.04** |
| Retired | 1.08 ± 1.28 | 0.40 |

AFP= alpha foetoprotein, Ref = reference, SE= standard error of coefficients

**Table S5: Multivariate linear mixed-effects model of baseline factors associated with predicted death.**

| | β ± SE | p |
|---|---|---|
| Gender of the patient (Female) (ref : Male) | 3.57 ± 1.43 | **0.02** |
| Age of the patient (years old) | 0.07 ± 0.05 | 0.14 |
| Non tumor fibrosis F2/F3 (ref : F0/F1) | 0.64 ± 2.08 | 0.76 |
| Non tumor fibrosis F4 | -1.37 ± 1.36 | 0.31 |
| BCLC C (ref : BCLC B) | -2.48 ± 1.21 | **0.04** |
| Child Pugh score – B (ref : A) | -1.87 ± 1.30 | 0.15 |
| Serum AFP level (logarithmic) | -0.39 ± 0.15 | **0.01** |
| Performance status – 1/2 (ref : 0) | -2.95 ± 1.04 | **0.005** |
| Previous treatment for HCC – Yes (ref : No) | 1.11 ± 1.06 | 0.29 |
| Numbers of patients treated for HCC each year by the doctor between 50 and 100 (ref ≤ 50) | 2.97 ± 2.01 | 0.15 |
| Numbers of patients treated for HCC each year by the doctor > 100 | 1.49 ± 2.14 | 0.49 |

AFP= alpha foetoprotein, BCLC = Barcelona clinic cancer, HCC = hepatocellular carcinoma, Ref = reference, SE= standard error of coefficients

**Table S6: Baseline factors associated with observed death using Cox proportional hazards model**

| | **HR [95%CI]** | **p** |
|---|---|---|
| Country – France (ref : Belgium) | 0.80 [0.50-1.30] | 0.37 |
| BCLC C (ref : BCLC B) | 1.91 [1.16-3.13] | **0.01** |
| Child Pugh score – B (ref : A) | 1.86 [1.15-3.01] | **0.01** |
| Serum AFP level (logarithmic) | 1.03 [0.97-1.09] | 0.36 |
| Performance status – 1/2 (ref : 0) | 1.67 [1.12-2.47] | **0.01** |
| Type of treatment – Sorafenib | 22.77 [5.08-102.12] | < **0.001** |
| HADS score total | 1.02 [0.99-1.05] | 0.26 |
| Married, in a relationship (ref: Single) | 0.65 [0.38-1.13] | 0.13 |
| Divorced, separated | 1.40 [0.68-2.89] | 0.36 |
| Widowed | 0.91 [0.42-1.96] | 0.81 |

AFP= alpha foetoprotein, BCLC = Barcelona clinic cancer, HADS = Hospital Anxiety and Depression Scale, HR= Hazard Ratio, 95% CI = 95% confidence interval

# Table S7: correlation between results of HADS survey with the answers to the patient's survey using multivariate linear regression

| | Total | β ± SE | P value |
|---|---|---|---|
| | N= 205 patients | | |
| **Socio-cultural level and lifestyle** | | | |
| **1. What is your marital/family status?** | | | |
| Single | 26 (13.0%) | reference | reference |
| Married, in a relationship | 130 (65.0%) | -0.127 ± 1.363 | > 0,9 |
| Divorced, separated | 23 (11.5%) | 0.785 ± 1.832 | 0.67 |
| Widowed | 21 (10.5%) | 1.493 ± 1.857 | 0.42 |
| **2. At home, do you live?** | | | |
| Alone | 47 (23.5%) | reference | reference |
| With someone constantly | 141 (70.5%) | -1.029 ± 1.081 | 0.34 |
| With someone intermittently present | 12 (6.0%) | -1.666 ± 2.101 | 0.43 |
| **3. Regarding your education, what is the highest level of your degree?** | | | |
| Primary school | 47 (24.0%) | | |
| High school : general education | 52 (26.5%) | 1.253 ± 1.309 | 0.34 |
| High school : vocational education | 61 (31.1%) | 0.347 ± 1.224 | 0.78 |
| University or higher education | 36 (18.4%) | -0.955 ± 1.392 | 0.49 |
| **4. What is your current employment status?** | | | |
| Active worker | 32 (16.0%) | | |
| Unemployed (Job seeker) | 11 (5.5%) | 2.708 ± 2.218 | 0.22 |
| Unable to work, disabled | 20 (10.0%) | 3.676 ± 1.841 | 0.047 |
| Retired | 137 (68.5%) | 0.795 ± 1.285 | 0.54 |
| **5. What is your current alcohol consumption?** | | | |
| Every day/every week | 29 (14.5%) | | |
| Occasional (less than once per week) | 39 (19.5%) | 0.43 ± 1.575 | 0.78 |
| Never | 132 (66.0%) | -0.094 ± 1.332 | > 0,9 |
| **Consultation for the announcement of cancer's treatment** | | | |
| **1. Did you have enough time to discuss your illness with your doctor? * %** | | | |
| Yes, completely | 150 (74.6%) | reference | reference |
| Mostly yes | 44 (21.9%) | 2.258 ± 1.077 | 0.04 |
| Mostly no + no, not at all | 7 (3.5%) | 3.344 ± 2.34 | 0.15 |
| **2. Did the doctor explain the type of treatment you will receive in a way that was understandable to you? * %** | | | |
| Yes, completely | 172 (85.6%) | reference | reference |
| Mostly yes | 25 (12.4%) | 2.229 ± 1.327 | 0.09 |
| Mostly no + no, not at all | 4 (2.0%) | 5.734 ± 2.97 | 0.05 |
| **3. Did the doctor listen to what you had to say? * %** | | | |
| Yes, completely | 177 (88.1%) | reference | reference |
| Mostly yes | 23 (11.4%) | 1.633 ± 1.377 | 0.24 |
| Mostly no + no, not at all | 1 (0.5%) | 0.875 ± 5.774 | 0.88 |
| **4. Did the doctor discuss the possibility of treatment-related side effects with you? * %** | | | |
| Yes, in detail | 161 (80.5%) | reference | reference |
| Yes, briefly | 29 (14.5%) | 0.561 ± 1.25 | 0.65 |
| No, not at all | 10 (5.0%) | 3.992 ± 1.9 | 0.04 |
| **5. Did the doctor discuss your life expectancy with you? * %** | | | |
| Yes, in detail | 37 (18.7%) | reference | reference |
| Yes, briefly | 43 (21.7%) | 1.201 ± 1.436 | 0.40 |
| No, not at all | 118 (59.6%) | -0.409 ± 1.225 | 0.74 |
| **6. Do you wish to have specific numerical information (in terms of months or percentage) about your life expectancy?** | | | |
| Yes | 78 (39.0%) | reference | reference |
| No | 80 (40.0%) | -3.654 ± 0.99 | < 0,001 |
| I don't know | 42 (21.0%) | -2.598 ± 1.168 | 0.03 |
| **7. Were you involved as much as you wanted in the choice of the proposed treatment?*** | | | |
| Yes, completely | 104 (52.0%) | reference | reference |
| Mostly yes | 74 (37.0%) | 0.502 ± 0.954 | 0.60 |
| Mostly no | 15 (7.5%) | 3.077 ± 1.731 | 0.08 |

| | | | |
|---|---|---|---|
| No, not at all | 6 (3.0%) | 2.519 ± 2.467 | 0.31 |
| I did not wish to be involved | 1 (0.5%) | 4.595 ± 5.675 | 0.42 |
| **8. Overall, how do you evaluate the quality of your consultation?*** | | | |
| Excellent | 108 (54.3%) | reference | reference |
| Very good | 81 (40.7%) | 1.445 ± 0.937 | 0.12 |
| Average | 9 (4.5%) | 2.313 ± 1.939 | 0.23 |
| Poor + very poor | 1 (0.5%) | -1.572 ± 5.657 | 0,78 |
| **9. Do you trust your doctor's judgment regarding the proposed treatment?* #** | | | |
| Yes, completely | 171 (85.5%) | reference | reference |
| Mostly yes | 28 (14.0%) | 4.151 ± 1.232 | 0.0009 |
| Mostly no + no, not at all | 1 (0.5%) | 0.487 ± 5.392 | > 0,9 |
| **Understanding of the disease** | | | |
| **1. How do you assess your current health status?** | | | |
| I feel healthy despite my illness, and I hope to recover | 125 (62.8%) | reference | reference |
| I feel healthy despite my illness, but I won't be able to recover | 43 (21.6%) | 3.352 ± 1.033 | 0.001 |
| I feel very sick due to my illness, but I hope to recover | 21 (10.6%) | 6.362 ± 1.407 | < 0,001 |
| I feel very sick due to my illness, and I won't be able to recover | 6 (3.0%) | 9.247 ± 2.409 | 0.0002 |
| I have no opinion | 4 (2.0%) | 6.353 ± 2.944 | 0.03 |
| **2. In your opinion, what is the stage of your cancer?** | | | |
| I do not have cancer | 2 (1.0%) | reference | reference |
| Early, very localized cancer | 27 (13.5%) | 0.319 ± 4.613 | > 0,9 |
| Intermediate stage cancer | 64 (32.0%) | 1.742 ± 4.519 | 0.70 |
| Advanced stage cancer | 56 (28.0%) | 2.819 ± 4.528 | 0.53 |
| I don't know | 51 (25.5%) | 2.313 ± 4.544 | 0.61 |
| **3. In your opinion, what could be the impact of the illness in terms of life expectancy? %** | | | |
| I have a life expectancy of more than 5 years | 100 (63.3%) | reference | reference |
| I have a life expectancy of 2 to 5 years | 37 (23.4%) | 2.04 ± 1.149 | 0.08 |
| I have a life expectancy of less than 2 years | 21 (13.3%) | 3.14 ± 1.354 | 0.02 |
| **Understanding of the treatment** | | | |
| **1. Among the following options, which one best corresponds to what your doctor told you during the consultation regarding your treatment– N(%)** | | | |
| My cancer will be cured | 15 (7.5%) | reference | reference |
| My cancer can be cured if the treatment works | 89 (44.5%) | 3.683 ± 1.739 | 0.04 |
| My cancer cannot be cured, but we will try to control the disease with treatment | 70 (35.0%) | 5.397 ± 1.779 | 0.003 |
| I don't know | 15 (7.5%) | 6.453 ± 2.006 | 0.002 |
| **2. What percentage chance do you believe there is that your treatment will shrink or stop the progression of your cancer? %** | | | |
| Between 0 and 25% | 13 (7.3%) | reference | reference |
| Between 25 and 50% | 41 (23.0%) | 1.64 ± 2.241 | 0.47 |
| Between 50 and 75% | 50 (28.1%) | 0.503 ± 1.994 | 0.80 |
| Between 75 and 100% | 43 (24.2%) | -0.748 ± 2.072 | 0.72 |
| 100% | 31 (17.4%) | -2.217 ± 2.22 | 0,32 |
| **3. Is maintaining your quality of life more important to you than living longer?– N(%)** | | | |
| Yes, completely | 73 (37.2%) | reference | reference |
| Mostly yes | 81 (41.3%) | 1.725 ± 1.009 | 0.09 |
| Mostly no | 31 (15.8%) | 0.225 ± 1.351 | 0.87 |
| No, not at all | 11 (5.6%) | -2.175 ± 2.035 | 0.29 |
| **4. What percentage chance do you believe there is that the treatment will cause disabling side effects (such as diarrhea, vomiting, significant fatigue, pain...)?– N(%)%** | | | |
| 0% | 16 (8.7%) | reference | reference |
| Between 0 and 25% | 64 (35.0%) | 1.856 ± 1.719 | 0.28 |
| Between 25 and 50% | 71 (38.8%) | 4.354 ± 1.687 | 0.01 |
| Between 50 and 75% | 28 (15.3%) | 3.654 ± 1.928 | 0.06 |
| Between 75 and 100% | 4 (2.1%) | 7.591 ± 3.574 | 0.04 |
| **5. From how many months of life expectancy gain are you willing to accept significant side effects (such as nausea/vomiting, significant fatigue, or pain)?– N(%)%** | | | |
| 0 to 3 months | 37 (22.4%) | reference | reference |
| 3 to 6 months | 21 (12.7%) | 1.335 ± 1.669 | 0.43 |
| 6 to 12 months | 28 (17.0%) | 0.006 ± 1.611 | > 0,9 |
| 12 to 24 months | 25 (15.2%) | 0.401 ± 1.648 | 0.81 |
| > 24 months | 54 (32.7%) | 0.267 ± 1.29 | 0.84 |

| | | | |
|---|---|---|---|
| **6. I would like to try treatments for my cancer if they can make me live longer, even if it is very likely that they:** | | | |
| **6.1- Present a high level of side effects (such as nausea/vomiting, significant fatigue, or pain)– N(%)** | | | |
| Completely agree | 47 (23.9%) | reference | reference |
| Agree | 76 (38.6%) | 1.379 ± 1.154 | 0.23 |
| Disagree | 27 (13.7%) | 2.373 ± 1.508 | 0.12 |
| Strongly disagree | 7 (3.6%) | 0.685 ± 2.532 | 0.79 |
| No opinion | 40 (20.3%) | -1.509 ± 1.323 | 0.26 |
| **6.2- Require me to be bedridden and render me unable to use the bathroom or toilet without assistance – N(%)** | | | |
| Completely agree | 15 (7.8%) | reference | reference |
| Agree | 27 (14.0%) | 3.469 ± 2.018 | 0.09 |
| Disagree | 59 (30.6%) | 2.579 ± 1.775 | 0.15 |
| Strongly disagree | 54 (28.0%) | 4.555 ± 1.842 | 0.01 |
| No opinion | 38 (19.7%) | 1.962 ± 1.895 | 0.30 |
| **6.3- Require me to rely on help from my family and friends to carry out daily activities (such as shopping and managing money)– N(%)** | | | |
| Completely agree | 29 (15.0%) | reference | reference |
| Agree | 60 (31.1%) | 1.22 ± 1.402 | 0.39 |
| Disagree | 39 (20.2%) | -0.468 ± 1.494 | 0.75 |
| Strongly disagree | 36 (18.7%) | 2.758 ± 1.606 | 0.09 |
| No opinion | 29 (15.0%) | -1.137 ± 1.646 | 0.49 |
| **6.4- Impair my memory or concentration – N(%)** | | | |
| Completely agree | 15 (7.9%) | reference | reference |
| Agree | 44 (23.0%) | 2.941 ± 1.806 | 0.11 |
| Disagree | 56 (29.3%) | 2.515 ± 1.768 | 0.16 |
| Strongly disagree | 33 (17.3%) | 4.632 ± 1.892 | 0.02 |
| No opinion | 43 (22.5%) | 1.233 ± 1.847 | 0.51 |
| **6.5- Make me occasionally confused and disoriented – N(%)** | | | |
| Completely agree | 16 (8.6%) | reference | reference |
| Agree | 40 (21.4%) | 4.042 ± 1.823 | 0.03 |
| Disagree | 51 (27.3%) | 2.089 ± 1.763 | 0.24 |
| Strongly disagree | 34 (18.2%) | 3.204 ± 1.902 | 0.09 |
| No opinion | 46 (24.6%) | 1.515 ± 1.802 | 0.40 |

We regroup some categories of answers for the analysis when the numbers
