# Supplementary material for: Patient and physician expectations regarding disease and treatment of advanced HCC: The prospective PERCEPTION1 study
Source: JHEP Rep. 2024 Aug 22;6(11):101192. doi: 10.1016/j.jhepr.2024.101192 (PMC11686048; doi:10.1016/j.jhepr.2024.101192)
Supplement: Multimedia component 4 [file mmc4.pdf]

Patient and physician expectations regarding disease and treatment of advanced HCC: The prospective PERCEPTION1 study

Jean-Charles Nault[1,2,*], Nanthara Sritharan[3], Gontran Verset[4], Ivan Borbath[5], Marie Lequoy[6], Manon Allaire[7], Hélène Regnault[8], Isabelle Colle[9], Hans Orlent[10], Isabelle Sinapi[11], Christophe Moreno[12,13], Edouard Larrey[7], Sabrina Sidali[2,14], Clémence Hollande[14], Giuliana Amaddeo[8], Stanislas Pol[15], Pierre Nahon[1,2], Nathalie Ganne-Carrié[1,2], Vincent Levy[3], Coralie Bloch-Queyrat[3], *Paris Liver Cancer Group*, Eric Trepo[12,13], Mohammed Bouattour[14]

JHEP Reports **2024**. vol. 6 | 1–11

Check for updates

Background & Aims: We aimed to explore patient expectations regarding their treatments and prognosis in comparison to physicians' assessments in patients with advanced hepatocellular carcinoma (HCC) receiving systemic treatments.

Methods: We prospectively enrolled 205 patients in France and Belgium with Barcelona Clinic Liver Cancer (BCLC) stage B/C HCC receiving systemic treatment (NCT04823754). Patients completed a 28-question survey and the hospital anxiety and depression scale (HADS), while physicians filled a 17-question survey after the initial consultation. Univariate and multivariate models were used to assess factors associated with concordant patient-physician responses, HADS, as well as predicted (by physicians) and observed overall survival.

Results: Patients had a median age of 68 years with 75% having BCLC C HCC; 86.3% received atezolizumab/bevacizumab. 60% of patients did not discuss life expectancy with the physician. 63% of the patients believed they had a life expectancy >5 years. Among shared questions between patients and physicians, 36.4% concordance was observed; major differences centered on life expectancy with patients more optimistic than physicians. A lower patient-physician concordance was seen with shorter-consultations (p = 0.003), female physicians (p = 0.02), BCLC C (p = 0.03) and >100 HCC patients/year per physician (p = 0.008). Compared to France, patients from Belgium were more likely to be satisfied with the consultation (p <0.001) but were less optimistic about life expectancy. Using HADS, 52% of the patients had anxiety/depression that was correlated with alpha-fetoprotein level (p = 0.03). The predicted median overall survival by physicians was 18 months *vs*. 13 months for the observed overall survival (weak correlation, ρ = 0.31).

Conclusion: Expectations regarding systemic treatments for advanced HCC differ significantly between patients and physicians, showing notable variations across countries.

Clinical trial number: NCT04823754.

© 2024 The Author(s). Published by Elsevier B.V. on behalf of European Association for the Study of the Liver (EASL). This is an open access article under the CC BY license (http://creativecommons.org/licenses/by/4.0/).

Introduction

Advanced hepatocellular carcinoma (HCC) carries a poor prognosis.[1] Despite the approval of atezolizumab/bevacizumab or durvalumab/tremelimumab, the median overall survival remains 16 to 19 months.[2,3] Many patients experience disease progression during treatment, and achieving a cure for advanced HCC through systemic treatment remains infrequent.[4]

Previous research has centered on understanding patients' perception of the disease and prognosis in non-curative cancers, revealing a disparity between patient knowledge and the disease's limited prognosis.[5,6] Such discordance between physicians' and patients' expectations has also been described in other fields of medicine such as intensive care.[7] A comprehensive understanding of the disease and treatment outcomes is crucial to empower patients.[8,9] However, there is a scarcity of data specifically focusing on HCC. Moreover, few studies directly compare patients' and physicians' perspectives on prognosis and treatment effects. A significant debate revolves around how to communicate life expectancy with patients, an essential aspect of the physician-patient relationship.[10] Understanding and accepting potential treatment-related adverse events are also pivotal considerations. The relationship between patients and physicians forms the cornerstone of clinical care, warranting more research in advanced HCC.[11] A more comprehensive description of social and cultural factors across different countries is necessary to better understand how they influence the physician-patient relationship.

* Corresponding author. Address: Cordeliers research Center, AP-HP, Avicenne hospital, Hepatology unit, 125 rue de Stalingrad 93000 Bobigny, France.
E-mail address: naultjc@gmail.com (J.-C. Nault).
https://doi.org/10.1016/j.jhepr.2024.101192

Patients' understanding of the disease and the potential effect of the treatment and their relationship with their physician are important points in the clinical management of cancer. Studying these points could identify areas that need to be improved using targeted intervention. We performed a multicenter prospective study involving surveys completed by patients with advanced HCC and their physicians in France and Belgium to investigate patients' expectations regarding their treatments and prognosis compared to their physicians' assessments.

Materials and methods

Study design

We conducted a prospective multicentric study across six centers in France and five centers in Belgium (Clinical trial number NCT04823754) including patients between May 2021 and November 2023. All centers were tertiary centers expert in HCC with most of centers localized in the urban area. The promotion of the study was academic with funding from Ipsen who were not involved in the analysis of the results, nor the writing of the manuscript. All patients signed informed consent. National ethic committees in Belgium (P2020/448) and in France validated the study (IDRCB:2021-A00494-37).

The inclusion criteria were: patients >18 years old, Barcelona Clinic Liver Cancer (BCLC) B or C HCC diagnosed according to the EASL guidelines 2018,[12] undergoing systemic treatment (whatever the line of systemic treatment), with medical insurance, and performance status 0-2.

The exclusion criteria were, receiving neoadjuvant or adjuvant systemic treatment, or receiving a combination of systemic and locoregional treatments, such as trans-arterial chemoembolization and selective internal radiation therapy.

Sample size calculation

The percentage of concordance between physicians and patients is assumed to be within 25% to 50% based on clinical assumption. The sample size calculation was based on achieving a 95% CI around the average of these percentages. With a standard deviation of 10%, 170 patients will be required to achieve a precision around the average of approximately 1.5%. We decided to include 200 patients in order to have a significant power to perform subgroup analysis.

Baseline characteristics of the patients and physicians

At baseline, we recorded the demographic variables (age, gender, socioeconomic level), the delay between the first diagnosis of HCC and the inclusion in the study, the cause of the underlying liver disease, the degree of fibrosis of the nontumor liver, the Child-Pugh score, the serum alphafetoprotein (AFP) level, the performance status, the BCLC stage, the line of systemic treatment and the treatment received. On January 2024, the date of the last news and the status at the last news (dead or alive) was recorded.

The following data were recorded about all the physicians that participated in this study and filled the survey: gender, year of birth, medical specialty (medical oncology *vs.* hepatogastroenterology), year of obtainment of medical degree and number of patients with HCC treated per year.

Surveys

All the surveys were completed within 72 h following the initial consultation explaining the systemic treatment to the patient (after validation by a multidisciplinary tumor board).

All patients answered a survey including 28 questions: 5 questions to characterize the socio-cultural level of the patient and lifestyle, 10 questions about the feeling and feedback from the patient about the initial consultation, three questions about their understanding of the disease, 10 questions about the expectancy of the patient about the efficacy of the treatment and the potential adverse events related to the treatment, and three open questions: 1) Can you define your illness in a few words ? 2) "What are the expected outcomes of the treatment that has been prescribed to you? 3) How do you think your health condition will evolve over the next 12 months?

Among these 28 questions, 8 questions were used to assess the global satisfaction of the patients about the consultation.

All patients answered the hospital anxiety and depression scale (HADS) that assesses the level of anxiety (7 questions) and depression (7 questions) using a total of 14 questions on a 0 to 3-point Likert scale. The HADS score ranges from 0 to 21 and can be categorized into three categories (between 0 and 7 = no symptoms, between 8 to 10 = doubtful symptoms and 11 and more = certain symptoms).

Physicians answered a survey including 18 questions: nine questions about the feeling and feedback from the physician about the initial consultation, and nine questions about the expectancy of the physician about the efficacy of the treatment and the potential adverse events related to the treatment.

To note, 10 questions were in common between the physicians' and patients' survey to evaluate concordance in disease and treatment understanding between the patient and the physician.

We also asked all physicians to provide an estimation of overall survival (predicted survival) for each patient at the time of consultation in order to compare this prediction with the survival observed in clinical practice for each patient (observed survival).

Statistical analysis

The collected data were described using frequency and percentage for categorical variables and median (IQR) for quantitative variables. To compare the characteristics according to the country of origin, we applied Fisher's exact test or $\chi 2$ test, as appropriate, for categorical variables and Mann-Whitney test for quantitative variables. The estimation of the concordant response percentage between patient and physician was represented by a mean and its 95% CI. Univariate and multivariate generalized estimating equations with a Gaussian distribution were used to assess factors associated with the patient-physician concordant response percentage. An exchangeable correlation structure was chosen based on the quasi-likelihood under the independence model criterion (QIC). The β coefficient and SE were reported. Factors with a p value ≤0.1 in the univariate analysis were added to the multivariate model. Weighted Kappa coefficient was calculated to evaluate the degree of concordance about the perception of prognosis by the patient-physician and potential treatment side effects. To summarize the results of the three open-ended questions, the

authors manually recoded similar answers into categories. Patient satisfaction score about consultation was estimated using the same methodology as the percentage of concordant responses and a linear mixed-effects model was conducted to identify associated factors. For the total HADS score and its two dimensions (anxiety/depression), a linear model was performed. The β coefficient and SE were reported.

Overall survival was analyzed using univariate and multivariate Cox proportional hazards regression model. The hazard ratio and 95% CI were reported.

A linear mixed-effects model was also used to evaluate factors associated with predicted overall survival. To compare observed and predicted survival with country of origin a Log-rank test and Mann-Whitney test were performed, respectively. Spearman's correlation was used to assess correlation between observed survival and predicted survival.

Multiple imputation was performed using the multiple imputation chain equation method to account for missing data. The number of multiple imputations was set to 10 with 10 iterations. The results were aggregated by pooling the estimates obtained from each imputed dataset according to Rubin's rules. All statistical analyses were conducted using R software (version 3.6.1). p values less than 0.05 were considered indicative of statistical significance.

Results

Description of the population

A total of 205 patients were included in the study (150 in France and 55 in Belgium). Eighty-six percent were male with a median age of 68 years and 73.9% had cirrhosis (Table 1; Fig. S1). HCCs were classified as BCLC B in 25.5% of cases and BCLC C in 74.5% of cases. Eighty-five percent of the patients included received a first-line systemic treatment; with atezolizumab/bevacizumab for most of them in first line (91.4%). Twenty-eight different physicians (9 in Belgium and 19 in France) included patients in the study (Tables S1 and S2). The median length of the consultation was 30 min (IQR 25.0–35.0). During this consultation, the patient was accompanied by a family member or a friend in 51.5% of cases. A nurse was present together with the physician during the consultation in 22.8% of cases and after the consultation in 47% of cases.

Perception of the consultation, the disease and the treatment by patients

First, we focused on the answers of patients to the survey assessing their feedback on the consultation based on eight questions (Table 2). Most patients (82.4%; 95% CI 80.50–84.33) reported positive feedback on the consultation. In multivariate linear mixed-effects regression, the country (Belgium) ($\beta \pm SE = 8.79 \pm 2.76$) and use of atezolizumab/bevacizumab were associated with more positive feedback ($\beta \pm SE = 6.94 \pm 2.86$) about the consultation. Presence of a nurse during or after the consultation was not significantly associated with better satisfaction ($p = 0.70$). We did not identify any significant association between socio-cultural features of the patients (Table 2) and the degree of satisfaction.

Next, we assessed patients' understanding of the disease and the potential effect of treatment. No discussion about life expectancy occurred during the consultation in 59.6% of cases (Table 2). However, 39% of patients declared that they wanted numerical information about prognosis from their doctors, 40% did not want to have any prognosis duration and 21% did not

Table 1. Patient characteristics.

	Available data	Total N = 205	Belgium n = 55	France n = 150	*p* value
Age (years old)$	205	68.0 [62.0; 75.0]	68.0 [62.0; 76.5]	68.0 [61.0; 74.8]	0.83
Gender (male)*	204	176 (86.3%)	48 (88.9%)	128 (85.3%)	0.67
Chronic alcohol intake*	205	107 (52.2%)	34 (61.8%)	73 (48.7%)	0.13
Chronic hepatitis B*		17 (8.3%)	2 (3.6%)	15 (10.0%)	0.25
Chronic hepatitis C*		54 (26.3%)	13 (23.6%)	41 (27.3%)	0.72
MASLD*		69 (33.7%)	7 (12.7%)	62 (41.3%)	<0.001
Cirrhosis*	199	147 (73.9%)	40 (72.7%)	107 (74.3%)	0.9
BCLC stage B*	204	52 (25.5%)	25 (45.5%)	27 (18.1%)	<0.001
BCLC stage C*		152 (74.5%)	30 (54.5%)	122 (81.9%)	
Child-Pugh A*	193	163 (84.5%)	43 (82.7%)	120 (85.1%)	0.85
Serum AFP level$	182	51.5 [6.8; 1325.0]	85.2 [6.0; 662.0]	47.0 [7.1; 1512.0]	0.52
Performance status 0*	200	103 (51.5%)	32 (59.3%)	71 (48.6%)	0.22
Performance status 1*		85 (42.5%)	21 (38.9%)	64 (43.8%)	
Performance status 2*		12 (6.0%)	1 (1.9%)	11 (7.5%)	
Past history of treatment for HCC	204	120 (58.8%)	29 (52.7%)	91 (61.1%)	0.36
**Description of the systemic treatments received at inclusion**					
Sorafenib*	205	10 (4.9%)	4 (7.3%)	6 (4.0%)	0.46
Lenvatinib*		1 (0.5%)	0 (0%)	1 (0.7%)	—
Regorafenib*		3 (1.5%)	0 (0%)	3 (2.0%)	—
Cabozantinib*		1 (0.5%)	1 (1.8%)	0 (0%)	—
Atezolizumab + bevacizumab*		177 (86.3%)	39 (70.9%)	138 (92.0%)	<0.001
Other treatments*		13 (6.3%)	11 (20.0%)	2 (1.3%)	<0.001
First-line treatment*	205	174 (85.3%)	46 (83.6%)	128 (85.9%)	0.62
Second-line treatment*		23 (11.3%)	6 (10.9%)	17 (11.4%)	
Third-line or more treatment*		7 (3.4%)	3 (5.5%)	4 (2.7%)	

AFP, alpha-fetoprotein; BCLC, Barcelona Clinical Liver Cancer; HCC, hepatocellular carcinoma; MASLD, metabolic associated steatotic liver disease. We applied Fisher's exact test or χ^2 test, as appropriate, for categorical variables and Mann-Whitney test for quantitative variables.

*Numbers (percentage).

$Median (interquartile range).

Table 2. Results of the patient survey.

	Total N = 205 patients	Belgium n = 55 patients	France n = 150 patients	*p* values	Available data
**Survey completed**	202 (98.5%)	55 (100.0%)	147 (98.0%)	—	205
**Socio-cultural level and lifestyle**					
1. What is your marital/family status?					
Single	26 (13.0%)	6 (10.9%)	20 (13.8%)	0.89	200
Married, in a relationship	130 (65.0%)	38 (69.1%)	92 (63.4%)		
Divorced, separated	23 (11.5%)	6 (10.9%)	17 (11.7%)		
Widowed	21 (10.5%)	5 (9.1%)	16 (11.0%)		
2. At home, do you live?					
Alone	47 (23.5%)	10 (18.2%)	37 (25.5%)	0.39	200
With someone constantly	141 (70.5%)	43 (78.2%)	98 (67.6%)		
With someone intermittently present	12 (6.0%)	2 (3.6%)	10 (6.9%)		
3. Regarding your education, what is the highest level of your degree?					
Primary school	47 (24.0%)	9 (16.7%)	38 (26.8%)	0.49	196
High school: general education	52 (26.5%)	15 (27.8%)	37 (26.1%)		
High school: vocational education	61 (31.1%)	18 (33.3%)	43 (30.3%)		
University or higher education	36 (18.4%)	12 (22.2%)	24 (16.9%)		
4. What is your current employment status?					
Active worker	32 (16.0%)	9 (16.4%)	23 (15.9%)	>0.9	200
Unemployed (Job seeker)	11 (5.5%)	3 (5.5%)	8 (5.5%)		
Unable to work, disabled	20 (10.0%)	6 (10.9%)	14 (9.7%)		
Retired	137 (68.5%)	37 (67.3%)	100 (69.0%)		
5. What is your current alcohol consumption?					
Every day	25 (12.5%)	5 (9.1%)	20 (13.8%)	0.07	200
Every week	4 (2.0%)	3 (5.5%)	1 (0.7%)		
Occasional (less than once per week)	39 (19.5%)	7 (12.7%)	32 (22.1%)		
Never	132 (66.0%)	40 (72.7%)	92 (63.4%)		
**Consultation for the announcement of cancer's treatment**					
1. When was your first appointment with the doctor you just saw?					
Today	63 (31.5%)	15 (27.3%)	48 (33.1%)	0.16	200
Less than a month ago	48 (24.0%)	15 (27.3%)	33 (22.8%)		
Between 1 and 3 months ago	14 (7.0%)	7 (12.7%)	7 (4.8%)		
Between 3 months and 1 year ago	31 (15.5%)	10 (18.2%)	21 (14.5%)		
More than a year ago	44 (22.0%)	8 (14.5%)	36 (24.8%)		
2. Did you have enough time to discuss your illness with your doctor?* %					
Yes, completely	150 (74.6%)	46 (83.6%)	104 (71.2%)	0.27	201
Mostly yes	44 (21.9%)	9 (16.4%)	35 (24.0%)		
Mostly no	5 (2.5%)	0 (0%)	5 (3.4%)		
No, not at all	2 (1.0%)	0 (0%)	2 (1.4%)		
3. Did the doctor explain the type of treatment you will receive in a way that was understandable to you?* %					
Yes, completely	172 (85.6%)	51 (92.7%)	121 (82.9%)	0.20	201
Mostly yes	25 (12.4%)	4 (7.3%)	21 (14.4%)		
Mostly no	4 (2.0%)	0 (0%)	4 (2.7%)		
No, not at all	0 (0%)	0 (0%)	0 (0%)		
4. Did the doctor listen to what you had to say?* %					
Yes, completely	177 (88.1%)	49 (89.1%)	128 (87.7%)	> 0.9	201
Mostly yes	23 (11.4%)	6 (10.9%)	17 (11.6%)		
Mostly no	1 (0.5%)	0 (0%)	1 (0.7%)		
No, not at all	0 (0%)	0 (0%)	0 (0%)		
5. Did the doctor discuss the possibility of treatment-related side effects with you?* %					
Yes, in detail	161 (80.5%)	48 (87.3%)	113 (77.9%)	0.11	200
Yes, briefly	29 (14.5%)	7 (12.7%)	22 (15.2%)		
No, not at all	10 (5.0%)	0 (0%)	10 (6.9%)		
6. Did the doctor discuss your life expectancy with you?* %					
Yes, in detail	37 (18.7%)	17 (31.5%)	20 (13.9%)	<0.001	198
Yes, briefly	43 (21.7%)	17 (31.5%)	26 (18.1%)		
No, not at all	118 (59.6%)	20 (37.0%)	98 (68.1%)		
7. Do you wish to have specific numerical information (in terms of months or percentage) about your life expectancy?					
Yes	78 (39.0%)	22 (40.0%)	56 (38.6%)	0.17	200
No	80 (40.0%)	26 (47.3%)	54 (37.2%)		
I don't know	42 (21.0%)	7 (12.7%)	35 (24.1%)		
8. Were you involved as much as you wanted in the choice of the proposed treatment?*					
Yes, completely	104 (52.0%)	34 (61.8%)	70 (48.3%)	0.04	200
Mostly yes	74 (37.0%)	19 (34.5%)	55 (37.9%)		
Mostly no	15 (7.5%)	1 (1.8%)	14 (9.7%)		
No, not at all	6 (3.0%)	0 (0%)	6 (4.1%)		
I did not wish to be involved	1 (0.5%)	1 (1.8%)	0 (0%)		

(*continued on next page*)

Table 2. (*continued*)

	Total N = 205 patients	Belgium n = 55 patients	France n = 150 patients	*p* values	Available data
9. Overall, how do you evaluate the quality of your consultation?*					
Excellent	108 (54.3%)	32 (59.3%)	76 (52.4%)	0.08	199
Very good	81 (40.7%)	21 (38.9%)	60 (41.4%)		
Average	9 (4.5%)	0 (0%)	9 (6.2%)		
Poor	1 (0.5%)	1 (1.9%)	0 (0%)		
Very poor	0 (0%)	0 (0%)	0 (0%)		
10. Do you trust your doctor's judgment regarding the proposed treatment?* %					
Yes, completely	171 (85.5%)	52 (94.5%)	119 (82.1%)	0.04	200
Mostly yes	28 (14.0%)	3 (5.5%)	25 (17.2%)		
Mostly no	1 (0.5%)	0 (0%)	1 (0.7%)		
No, not at all	0 (0%)	0 (0%)	0 (0%)		
**Understanding of the disease**					
1. How do you assess your current health status?					
I feel healthy despite my illness, and I hope to recover	125 (62.8%)	35 (63.6%)	90 (62.5%)	0.34	199
I feel healthy despite my illness, but I won't be able to recover	43 (21.6%)	14 (25.5%)	29 (20.1%)		
I feel very sick due to my illness, but I hope to recover	21 (10.6%)	4 (7.3%)	17 (11.8%)		
I feel very sick due to my illness, and I won't be able to recover	6 (3.0%)	0 (0%)	6 (4.2%)		
I have no opinion	4 (2.0%)	2 (3.6%)	2 (1.4%)		
2. In your opinion, what is the stage of your cancer?					
I do not have cancer	2 (1.0%)	1 (1.8%)	1 (0.7%)	0.38	200
Early, very localized cancer	27 (13.5%)	7 (12.7%)	20 (13.8%)		
Intermediate stage cancer	64 (32.0%)	22 (40.0%)	42 (29.0%)		
Advanced stage cancer	56 (28.0%)	15 (27.3%)	41 (28.3%)		
Terminal stage cancer	0 (0%)	0 (0%)	0 (0%)		
I don't know	51 (25.5%)	10 (18.2%)	41 (28.3%)		
3. In your opinion, what could be the impact of the illness in terms of life expectancy? %					
I have a life expectancy of more than 5 years	100 (63.3%)	22 (48.9%)	78 (69.0%)	0.03	158
I have a life expectancy of 2 to 5 years	37 (23.4%)	12 (26.7%)	25 (22.1%)		
I have a life expectancy of 1 to 2 years	18 (11.4%)	9 (20.0%)	9 (8.0%)		
I have a life expectancy of less than 1 year	3 (1.9%)	2 (4.4%)	1 (0.9%)		
**Understanding of the treatment**					
1. Among the following options, which one best corresponds to what your doctor told you during the consultation regarding your treatment – n (%)					
My cancer will be cured	15 (7.5%)	2 (3.6%)	13 (9.0%)	<0.001	200
My cancer can be cured if the treatment works	89 (44.5%)	28 (50.9%)	61 (42.1%)		
My cancer cannot be cured, but we will try to control the disease with treatment	70 (35.0%)	25 (45.5%)	45 (31.0%)		
I don't know	15 (7.5%)	2 (3.6%)	13 (9.0%)		
2. What percentage chance do you believe there is that your treatment will shrink or stop the progression of your cancer? %					
0%	2 (1.1%)	2 (3.8%)	0 (0%)	0.008	178
Between 0 and 25%	11 (6.2%)	5 (9.6%)	6 (4.8%)		
Between 25 and 50%	41 (23.0%)	16 (30.8%)	25 (19.8%)		
Between 50 and 75%	50 (28.1%)	12 (23.1%)	38 (30.2%)		
Between 75 and 100%	43 (24.2%)	14 (26.9%)	29 (23.0%)		
100%	31 (17.4%)	3 (5.8%)	28 (22.2%)		
3. Is maintaining your quality of life more important to you than living longer?– n (%)					
Yes, completely	73 (37.2%)	20 (36.4%)	53 (37.6%)	0.19	196
Mostly yes	81 (41.3%)	18 (32.7%)	63 (44.7%)		
Mostly no	31 (15.8%)	13 (23.6%)	18 (12.8%)		
No, not at all	11 (5.6%)	4 (7.3%)	7 (5.0%)		
4. What percentage chance do you believe there is that the treatment will cause disabling side effects (such as diarrhea, vomiting, significant fatigue, pain …)? – n (%)%					
0%	16 (8.7%)	2 (4.3%)	14 (10.3%)	0.27	183
Between 0 and 25%	64 (35.0%)	16 (34.0%)	48 (35.3%)		
Between 25 and 50%	71 (38.8%)	22 (46.8%)	49 (36.0%)		
Between 50 and 75%	28 (15.3%)	5 (10.6%)	23 (16.9%)		
Between 75 and 100%	1 (0.5%)	0 (0%)	1 (0.7%)		
100%	3 (1.6%)	2 (4.3%)	1 (0.7%)		
5. From how many months of life expectancy gain are you willing to accept significant side effects (such as nausea/vomiting, significant fatigue, or pain)? – n (%)%					
0 to 3 months	37 (22.4%)	9 (18.8%)	28 (23.9%)	0.88	165
3 to 6 months	21 (12.7%)	7 (14.6%)	14 (12.0%)		
6 to 12 months	28 (17.0%)	8 (16.7%)	20 (17.1%)		
12 to 24 months	25 (15.2%)	9 (18.8%)	16 (13.7%)		
>24 months	54 (32.7%)	15 (31.2%)	39 (33.3%)		

(*continued on next page*)

Table 2. (*continued*)

	Total N = 205 patients	Belgium n = 55 patients	France n = 150 patients	*p* values	Available data
6. I would like to try treatments for my cancer if they can make me live longer, even if it is very likely that they:					
6.1- Present a high level of side effects (such as nausea/vomiting, significant fatigue, or pain) – n (%)					
Completely agree	47 (23.9%)	16 (29.1%)	31 (21.8%)	0.55	197
Agree	76 (38.6%)	21 (38.2%)	55 (38.7%)		
Disagree	27 (13.7%)	7 (12.7%)	20 (14.1%)		
Strongly disagree	7 (3.6%)	3 (5.5%)	4 (2.8%)		
No opinion	40 (20.3%)	8 (14.5%)	32 (22.5%)		
6.2- Require me to be bedridden and render me unable to use the bathroom or toilet without assistance – n (%)					
Completely agree	15 (7.8%)	4 (7.3%)	11 (8.0%)	0.59	193
Agree	27 (14.0%)	9 (16.4%)	18 (13.0%)		
Disagree	59 (30.6%)	13 (23.6%)	46 (33.3%)		
Strongly disagree	54 (28.0%)	19 (34.5%)	35 (25.4%)		
No opinion	38 (19.7%)	10 (18.2%)	28 (20.3%)		
6.3- Require me to rely on help from my family and friends to carry out daily activities (such as shopping and managing money) – n (%)					
Completely agree	29 (15.0%)	11 (20.0%)	18 (13.0%)	0.46	193
Agree	60 (31.1%)	17 (30.9%)	43 (31.2%)		
Disagree	39 (20.2%)	10 (18.2%)	29 (21.0%)		
Strongly disagree	36 (18.7%)	12 (21.8%)	24 (17.4%)		
No opinion	29 (15.0%)	5 (9.1%)	24 (17.4%)		
6.4- Impair my memory or concentration – n (%)					
Completely agree	15 (7.9%)	4 (7.4%)	11 (8.0%)	0.34	191
Agree	44 (23.0%)	17 (31.5%)	27 (19.7%)		
Disagree	56 (29.3%)	13 (24.1%)	43 (31.4%)		
Strongly disagree	33 (17.3%)	11 (20.4%)	22 (16.1%)		
No opinion	43 (22.5%)	9 (16.7%)	34 (24.8%)		
6.5- Make me occasionally confused and disoriented – n (%)					
Completely agree	16 (8.6%)	4 (7.3%)	12 (9.1%)	0.34	187
Agree	40 (21.4%)	12 (21.8%)	28 (21.2%)		
Disagree	51 (27.3%)	13 (23.6%)	38 (28.8%)		
Strongly disagree	34 (18.2%)	15 (27.3%)	19 (14.4%)		
No opinion	46 (24.6%)	11 (20.0%)	35 (26.5%)		

We applied Fisher's exact test or χ^2 test, as appropriate, for categorical variables.
*These 8 questions are used to assess the global satisfaction of the patients about the consultation.
%These 10 questions are used to assess the concordance between the patients and the physicians.

know. In terms of understanding of the disease and the efficacy of the treatments, 13.5% of patients thought they had an early-stage cancer and 25.5% did not know how to classify their disease stage. 23% of patients thought they had a life expectancy between 2 and 5 years and 63.3% a life expectancy of more than 5 years. Patients with HCC classified as BCLC B answered more frequently that they have a life expectancy of more than 5 years compared to patients with HCC classified as BCLC C (66.7% for BCLC B *vs.* 37.5% for BCLC C, $p = 0.006$). Among patients, 7.5% reported that they will be cured of the cancer and 44.5% that they will be cured of the cancer if the treatments work. Interestingly, patients who discussed life expectancy in detail with the doctor answered "my cancer will be cured" in 2.7% of cases, that "my cancer can be cured if the treatment works" in 59.5% of cases, that "my cancer cannot be cured but we will try to control the disease with treatment" in 35.1% of cases, or "I don't know" in 2.7% of cases. In contrast, patients who did not discuss life expectancy with the doctor answered that "my cancer will be cured" in 9.5% of cases, that "my cancer can be cured if the treatment works" in 41.4% of cases, that "my cancer cannot be cured but we will try to control the disease with treatment" in 28.4% of cases, or "I don't know" in 20.7% of cases ($p < 0.001$). Most of the patients thought they had more than a 50% chance that their cancer would be stabilized or reduced in size thanks to treatment: 100% of a chance in 17.4% of cases, 75 to 100% of a chance in 24.2% of cases and between 50% and 75% of a chance in 28.1% of cases. Finally, in terms of what could be accepted by patients regarding adverse events balanced to the potential efficiency of the treatment, most patients thought that maintaining quality of life was more important than maintaining longer life (yes completely 37.2% and mostly yes 41.3%) and patients would accept significant side effects if the life expectancy gain was 12 to 24 months in 15% of cases and more than 24 months in 32.7% of cases.

To the open question "Can you define your illness in a few words?" (answered by 190 patients), 118 patients declared they had a cancer and 42 patients a liver disease. To the open question "What are the expected outcomes of the treatment that has been prescribed to you?" (answered by 194 patients), 47 patients declared that the treatment would cure the disease, 22 patients that the treatment would lead to decrease in size of the tumor and 35 patients that the treatment will stabilize the disease. To the open question "How do you think your health condition will evolve over the next 12 months?" (answered by 192 patients), 110 patients answered that their health condition will improve, 12 patients answered that their health condition will be stabilized, 11 patients that their health condition will deteriorate and 47 patients that they do not know.

Anxiety and depression in patients with advanced HCC

The results of the HADS survey showed that 52.4% of patients had symptoms of anxiety and/or depression (Table S3). In

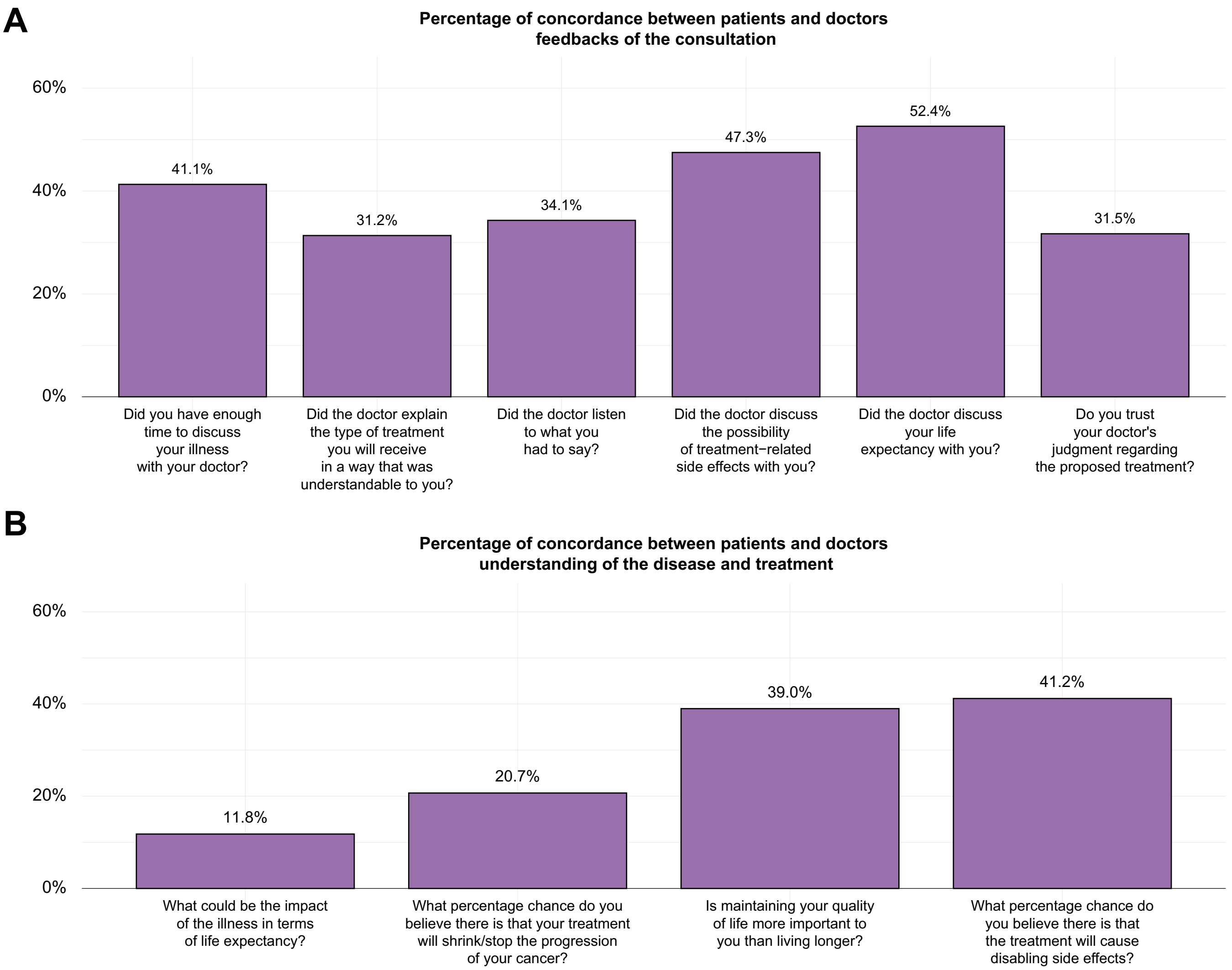

Fig. 1. Percentages of concordance between patients and physicians in questions in common across the surveys. We represented the % of concordance between patients and physicians for each of the 10 questions in common between both surveys.

multivariate analysis, serum AFP level (β ± SE = 0.34 ± 0.15, *p* = 0.03), as well as being unable to work or being disabled (β ± SE = 3.63 ± 1.81, *p* = 0.04), were the two variables independently positively associated with a high anxiety and depression score (Table S4). To note, serum AFP level was significantly associated with a higher rate of depression (β ± SE = 0.22 ± 0.08, *p* = 0.006) but not of anxiety (β ± SE = 0.12 ± 0.09, *p* = 0.19) defined by the HADS scale. Moreover, patients who answer "no" (β ± SE = -3.654 ± 0.99, *p* <0.001) to the question "Do you wish to have specific numerical information (in terms of months or percentage) about your life expectancy?" have a low anxiety and depression score. In contrast, patients who answered "I feel very sick due to my illness, and I won't be able to recover" (β ± SE = 9.247 ± 2.409, *p* = 0.0002) to the question "How do you assess your current health status?" and patients who answered "My cancer cannot be cured, but we will try to control the disease with treatment" (β ± SE = 5.397 ± 1.779, *p* = 0.003) to the question "which one best corresponds to what your doctor told you during the consultation regarding your treatment" had a high anxiety and depression score. Patients answering that they have less than 2 years of life expectancy have a higher anxiety and depression score (β ± SE = 3.14 ± 1.354, *p* = 0.02) (Table S7 for all the correlation between answers of the patient's answer and results of the HADS survey).

Concordance between physician and patient perception

We assessed the discrepancy between the perception of the patient and the physician in terms of prognosis of the disease and efficiency of the treatment using the 10 questions in common between the two surveys; results were available for 170 patients receiving a first-line treatment. The overall concordance of the answers between physician and patient was 36.41% (95% CI 33.53–39.30) in the whole population with a mean weighted kappa coefficient of 0.09 (95% CI -0.36 to 0.51) (weak correlation) (Fig. 1). The concordance was 38.12% (95% CI 32.16–44.09) in the 29 patients treated in the second or further lines. The concordance on answers to the question about the prediction of life expectancy was very low between patients and physicians with only 11.8% concordance in the first line-treated patients. In multivariate analysis, gender of the physician (female) and BCLC C stage and

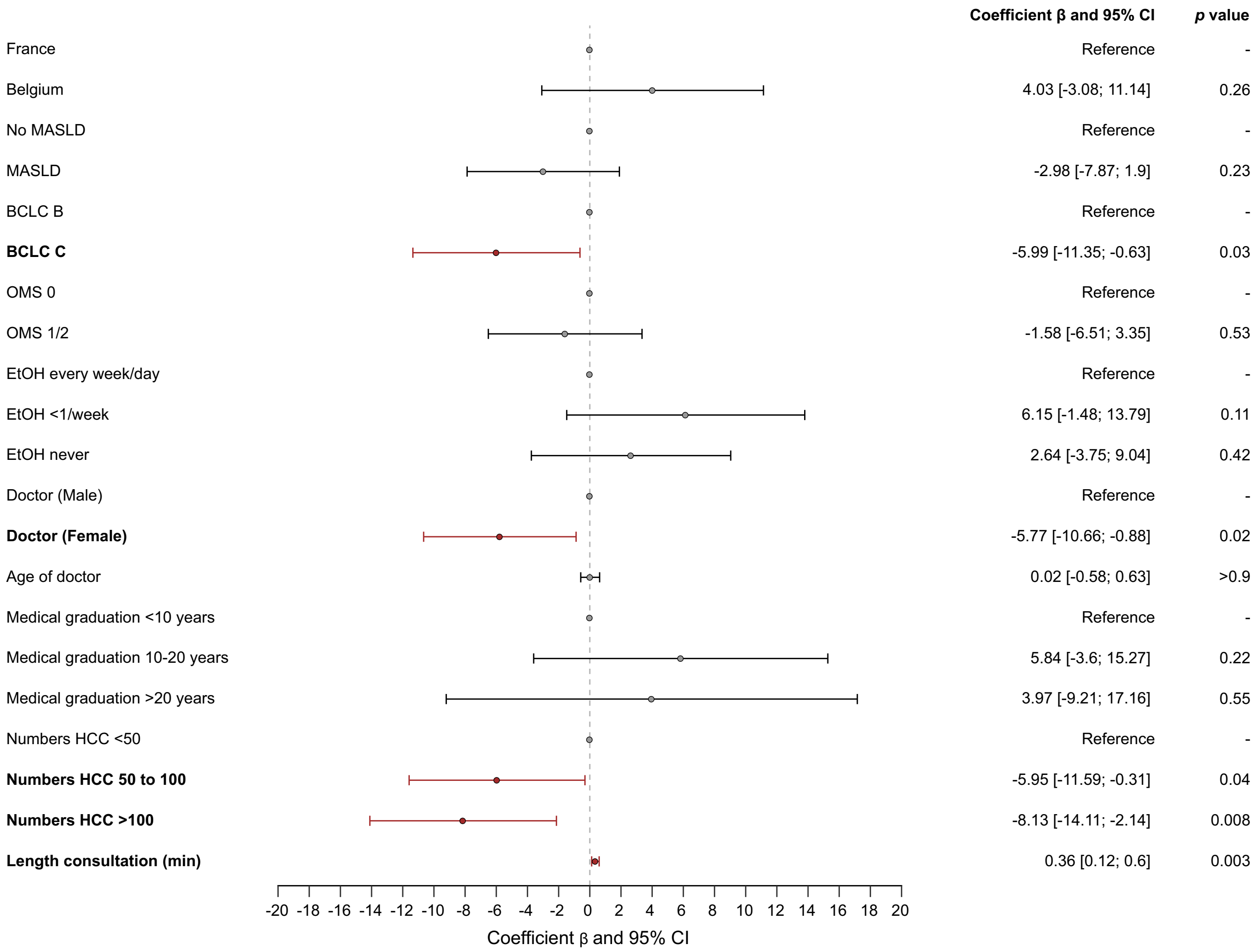

Fig. 2. Variables associated with concordance of answers between patients and physicians. We performed multivariate generalized estimating equations with a Gaussian distribution to assess factors associated with concordance of answers between patients and physicians. We used a forest plot depicting the multivariable generalized estimating equations showing the factors associated with concordance of answers between patients and physicians. BCLC, Barcelona Clinic Liver Cancer; EtOH, alcohol intake; MASLD, metabolic associated steatotic liver disease.

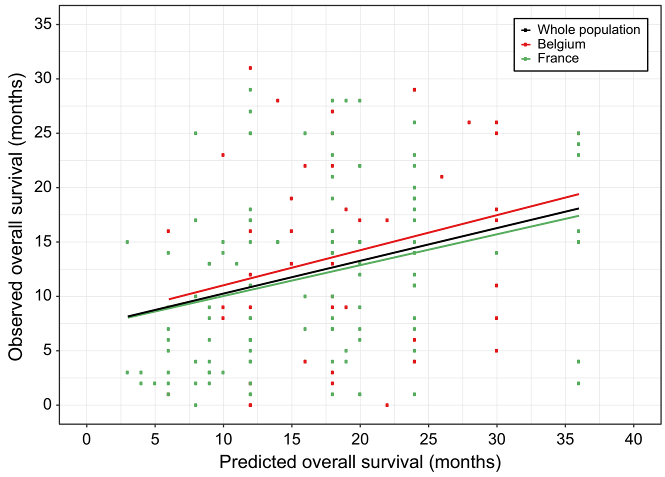

Fig. 3. Correlation between overall survival predicted by physicians and overall survival observed in clinical practice. We correlated the predicted overall survival by the physicians and the observed overall survival (overall survival) in patients (using Spearman ρ correlation) in the whole population (ρ = 0.31 [95% CI 0.16–0.44], in patients from Belgium (ρ = 0.26 [95% CI -0.07 to 0.50]) and in patients from France (ρ = 0.33 [95% CI 0.16–0.49]).

physicians who have seen a number of patients with HCC between 50 and 100 or more than 100 were associated with a lower concordance between physician and patient answers to the survey, whereas the length of consultation was associated with a higher concordance (Fig. 2). Presence of a nurse during or after the consultation was not significantly associated with a better concordance (p = 0.28). We did not identify any significant association between socio-cultural features of the patients and the concordance between patients and physicians (data not shown).

Difference between the answers to the survey between France and Belgium

Next, we compared the results obtained in France (150 patients) and Belgium (55 patients) (Table 2). The global satisfaction about the consultation was higher in Belgium (87.88%) than in France (80.38%, p <0.001). Patients reported that doctors discussed life expectancy more frequently in Belgium (31.5%) than in France (13.9%, p <0.001). In contrast, patients from Belgium thought that treatments would be less effective than patients from France in terms of chance of cure (p <0.001) or in terms of the ability to shrink or stop the cancer (p = 0.008). In terms of concordance between the answers to the survey

between the patient and the physician using the 10 questions in common between the two surveys, a mean concordance was observed in 44.78% (95% CI 38.90–50.66) of the cases in Belgium and 33.31% (95% CI 30.13–36.49) in France (p <0.001).

Comparison between the observed and predicted overall survival

Finally, we compared the prediction of survival for each patient provided by the physicians and the outcomes observed in clinical practice for these patients. The predicted median overall survival by the doctor was 18 months (95% CI 16–18) without any significant difference between France (18 months [95% CI 12–18]) and Belgium (18 months [95% CI 16–20], p = 0.1) (Fig. 3). Among the baseline variables from patients and doctors, gender of the patient (female) was associated with a longer predicted survival, whereas serum AFP level, performance status 1/2 and BCLC C stage were associated with a lower predicted survival (Table S5).

Among the 204 patients, the median follow-up was 23 months (95% CI 21–25). The median observed overall survival was 13 months (95% CI 11–16), with a 2-year survival of 33% [95% CI 26%–42%], without any significant difference between France (12 months [95% CI 9–15]) and Belgium (18 months [95% CI 12–NR], p = 0.08). Among the baseline patient variables, tumor stage, Child-Pugh score, performance status, and treatment with sorafenib were independently associated with risk of death (Table S6). To note, we observed no significant association between the answer to the question by the patients "In your opinion, what could be the impact of the illness in terms of life expectancy?" and the observed median survival (p = 0.8). We observed a weak correlation between the predicted survival and the observed survival as evidenced by Spearman correlation (ρ = 0.31 [95% CI 0.16–0.44]) in the whole population (Fig. 3). Using a margin of ±3 months, 52.3% of the patients lived less than the estimate, 23.9% lived up to the estimate and 23.9% of the patients lived more than the estimate.

Discussion

Herein, we report the results of prospective multicentric study focusing on the perception of the disease and treatment of patients with advanced HCC receiving systemic treatment, mainly atezolizumab/bevacizumab. First, patients reported to be globally satisfied by the consultation and the relationship with the physician. It is possible that some patients may have felt inclined to please their physician as the survey was filled immediately or in the days following the consultation, even though they were assured of anonymity.[13] In addition, there may be some bias related to the fact that some patients do not respond to certain questions, which could reduce the generalizability of the results. In contrast, one crucial aspect highlighted in the study is the necessity to better explain the stage of cancer to the patients because it is an important step to accurately understand the disease and better capture the potential effects of treatment. It was observed that a portion of the patients either had an incomplete understanding of their cancer stage or were unsure about it based on the results of the survey and of the open questions.

Another important point revealed by the study is there is a notable willingness among some patients to discuss their prognosis during the consultation. A majority of the patients saying that they want to prioritize maintaining a good quality of life over merely increasing survival.[14] This underscores also the importance of incorporating patient-reported outcomes using quality of life questionnaires developed for cancer and for HCC in randomized clinical trials.[15,16] More than half of the patients declared that they will be alive 5 years or more after the beginning of the treatment, underlining the important expectation in terms of treatment effect on their survival and in terms of the overall concept of curability as discussed in other cancer types.[17,18,19]However, 20% did not answer to the question about their life expectancy, indicating the potential difficulty in communicating or articulating certain aspects of their disease experience. A previous study mixing different types of metastatic solid cancers showed that most patients did not understand that systemic chemotherapy will not cure their disease.[5] In our study, the fact that the role of the nurse in patient management during or after the consultation was heterogeneous across centers and countries impaired granular analysis of their impact on patients' understanding of the disease. This highlights the need to provide more accurate information using therapeutic education programs with interprofessional work including dedicated nurses in order to promote shared decision-making with patients.[20]

Around 50% of the patients reported symptoms of anxiety and depression, underlining the necessity of psychological support in clinical practice. Furthermore, we identified an association between serum AFP levels and the presence of symptoms of anxiety and depression. This suggests that we must consider that regular monitoring of serum AFP levels could potentially affect the psychological well-being of patients, as suggested for PSA in prostate cancer.[21]

Moreover, we observed a significant lack of concordance between patients and physicians in terms of assessment of disease and treatment, particularly concerning the assessment of life expectancy with patients being more optimistic than their doctors. Interestingly, longer consultation durations were associated with better concordance, emphasizing the importance of thorough communication between patients and healthcare providers. Prediction of life expectancy and the effect of treatments is also difficult for physicians, as shown by the fact they are more optimistic in predicted survival compared to the observed survival of the patients. This disparity highlights the complex interplay between patient expectations, physician perspectives, and the reality of the disease trajectory.[22] Moreover, consultations had a median duration of 30 min, but previous studies have shown that a substantial amount of medical information given by doctors during consultations is forgotten immediately.[23]

There appears to be a divergence between Belgium and France in terms of patient satisfaction and concordance between physicians. Patients in Belgium reported higher satisfaction levels compared to their counterparts in France. Additionally, Belgian patients exhibited a more pessimistic outlook regarding their survival prospects. This difference could be potentially explained by cultural differences or disparities in management of patients with cancer between the two countries. Notably, there was more discussion on life expectancy among patients in Belgium, indicating potential differences in

communication practices. It is worth considering how these cultural and healthcare system factors might influence patient perceptions and experiences not only in these two countries but also in other regions globally.[17,24]

Finally, we did not observe any difference in our results in patients treated in second line or more *vs*. patients receiving a first-line treatment, potentially owing to the low numbers of patients treated in second line. Moreover, patients included in the second line are long-term survivors, and their responses could be influenced by survivorship bias. Another limitation of our study was the fact that we lacked longitudinal data to capture the dynamic nature of patients' experiences over time as we focused on only one moment in the disease course. Most of these patients have not yet experienced treatment and its side effects, which should be considered critical, as their expectations may change under treatment.[25]

In conclusion, our study has highlighted the disparities in expectations regarding systemic treatments for advanced HCC between patients and physicians, with significant variations observed between France and Belgium.

Affiliations

[1]*Liver unit, Avicenne hospital, APHP Bobigny, France;* [2]*Cordeliers research center, Sorbonne Université, Inserm, Université de Paris, team « Functional Genomics of Solid Tumors », Equipe labellisée Ligue Nationale Contre le Cancer, Labex OncoImmunology, F-75006 Paris, France;* [3]*URC-CRC GHPSS, Avicenne Hospital, Aphp Bobigny, France;* [4]*Gastrointestinal Oncology Unit, Hôpital Erasme, Université Libre de Bruxelles, Brussels, Belgium;* [5]*Department of Hepato-Gastroenterology, Cliniques Universitaires St Luc, Brussels, Belgium;* [6]*Liver Unit, Saint Antoine Hospital, APHP Paris, France;* [7]*Hepatogastroenterology department, hôpital Pitié Salpétrière, APHP, France;* [8]*Liver Unit, Henri Mondor Hospital, APHP Créteil, France;* [9]*Department of Gastroenterology and Hepatology, ASZ Aalst, Aalst, Belgium;* [10]*Department of Gastroenterology and Hepatology, AZ Sint Jan Brugge, Brugge, Belgium;* [11]*Department of Medical Oncology, GHdC-Grad Hopital de Charleroi-Site Notre Dame, 6000 Charleroi, Belgium;* [12]*Department of Gastroenterology, Hepatopancreatology and Digestive Oncology, Hôpital Universitaire de Bruxelles, Université Libre de Bruxelles, Brussels, Belgium;* [13]*Laboratory of Experimental Gastroenterology, Université Libre de Bruxelles, Brussels, Belgium;* [14]*Liver unit, Beaujon Hospital, APHP Clichy, France;* [15]*Liver unit, Cochin Hospital, APHP Paris, France*

Abbreviations

BCLC, Barcelona clinic liver cancer; HADS, hospital anxiety and depression scale; HCC, hepatocellular carcinoma.

Financial support

Funding support from Ipsen.

Conflict of interest

Jean-Charles Nault received research funding from Bayer and Ipsen. Stanislas Pol acted as speaker or board member for Janssen, Gilead, Abbvie, Novo-Nordisk, LFB, Pfizer, Vivv and received grants from Gilead and Abbvie. Christophe Moreno was paid as speaker or adviser from Astellas, Novartis, Bayer, Abbvie, Echosens, Surrozen, Intercept and Gilead Sciences pharmaceutical companies. He is consultant for Julius clinical. Nathalie Ganne-Carrie received travel and congress fees, consulting fees or honoraria for lectures, presentations, from Abbvie, Bayer, Gilead, Ipsen, Intercept and Roche. The other authors declare no COI.

Please refer to the accompanying ICMJE disclosure forms for further details.

Authors' contributions

Contributions to conception and design: JCN, VL, CBQ, ET, GV. Acquisition of data: JCN, NS, GV, IB, ML, MA, HR, IC, HO, IS, CM, EL, SS, CH, GA, SP, PN, NGC, ET, MB. Analysis and interpretation of data: JCN, NS, VL, CBQ, ET. Drafting, revising, and the manuscript content: JCN, NS, GV, VL, CBQ, ET, MB. Final approval of the version to be published: JCN, NS, GV, IB, ML, MA, HR, IC, HO, IS, CM, EL, SS, CH, GA, SP, PN, NGC, VL, CBQ, ET, MB.

Data availability statement

Data is available upon request from the corresponding author.

Role of the funding source

Ipsen provides a funding support for research but was not involve in data acquisition, statistical analysis, interpretation of data and manuscript writing.

Supplementary data

Supplementary data to this article can be found online at https://doi.org/10.1016/j.jhepr.2024.101192.

References

[1] Villanueva A. Hepatocellular carcinoma. N Engl J Med 2019;380:1450–1462.
[2] Finn RS, Qin S, Ikeda M, et al. Atezolizumab plus bevacizumab in unresectable hepatocellular carcinoma. N Engl J Med 2020;382:1894–1905.
[3] Abou-Alfa GK, Lau G, Kudo M, et al. Tremelimumab plus durvalumab in unresectable hepatocellular carcinoma. NEJM Evid 2022;1:EVIDoa2100070.
[4] Llovet JM, Pinyol R, Kelley RK, et al. Molecular pathogenesis and systemic therapies for hepatocellular carcinoma. Nat Cancer 2022;3:386–401.
[5] Weeks JC, Catalano PJ, Cronin A, et al. Patients' expectations about effects of chemotherapy for advanced cancer. N Engl J Med 2012;367:1616–1625.
[6] Lee EM, Jiménez-Fonseca P, Carmona-Bayonas A, et al. Clinicians' and advanced cancer patients' estimates of treatment efficacy and toxicity in oncologic treatment. Healthcare (Basel) 2023;11:2222.
[7] White DB, Ernecoff N, Buddadhumaruk P, et al. Prevalence of and factors related to discordance about prognosis between physicians and surrogate decision makers of critically ill patients. JAMA 2016;315:2086–2094.
[8] Sim JA, Shin JS, Park SM, et al. Association between information provision and decisional conflict in cancer patients. Ann Oncol 2015;26:1974–1980.
[9] Mack JW, Fasciano KM, Block SD. Communication about prognosis with adolescent and young adult patients with cancer: information needs, prognostic awareness, and outcomes of disclosure. J Clin Oncol 2018;36:1861–1867.
[10] Berger Z, Tung M, Yesantharao P, et al. Feasibility and perception of a question prompt list in outpatient cancer care. J Patient Rep Outcomes 2019;3:53.
[11] Soylu C, Babacan T, Sever AR, et al. Patients' understanding of treatment goals and disease course and their relationship with optimism, hope, and quality of life: a preliminary study among advanced breast cancer outpatients before receiving palliative treatment. Support Care Cancer 2016;24:3481–3488.
[12] European Association for the Study of the Liver. Electronic address: easloffice@easloffice.eu, European association for the study of the liver. EASL clinical practice guidelines: management of hepatocellular carcinoma. J Hepatol 2018;69:182–236.
[13] Kleeberg UR, Tews J-T, Ruprecht T, et al. Patient satisfaction and quality of life in cancer outpatients: results of the PASQOC study. Support Care Cancer 2005;13:303–310.
[14] Kehl KL, Landrum MB, Arora NK, et al. Association of actual and preferred decision roles with patient-reported quality of care: shared decision making in cancer care. JAMA Oncol 2015;1:50–58.
[15] Galle PR, Finn RS, Qin S, et al. Patient-reported outcomes with atezolizumab plus bevacizumab versus sorafenib in patients with unresectable hepatocellular carcinoma (IMbrave150): an open-label, randomised, phase 3 trial. Lancet Oncol 2021;22:991–1001.
[16] Jarnagin JX, Saraf A, Baiev I, et al. Patient-reported outcomes, tumor markers, and survival outcomes in advanced GI cancer. JAMA Netw Open 2023;6:e2343512.
[17] Yennurajalingam S, Rodrigues LF, Shamieh O, et al. Perception of curability among advanced cancer patients: an international collaborative study. Oncologist 2018;23:501–506.
[18] Loh KP, Mohile SG, Epstein RM, et al. Willingness to bear adversity and beliefs about the curability of advanced cancer in older adults. Cancer 2019;125:2506–2513.
[19] Epstein AS, Prigerson HG, O'Reilly EM, et al. Discussions of life expectancy and changes in illness understanding in patients with advanced cancer. J Clin Oncol 2016;34:2398–2403.

[20] Epstein RM, Duberstein PR, Fenton JJ, et al. Effect of a patient-centered communication intervention on oncologist-patient communication, quality of life, and health care utilization in advanced cancer: the VOICE randomized clinical trial. JAMA Oncol 2017;3:92–100.

[21] Lofters A, Juffs HG, Pond GR, et al. PSA-itis': knowledge of serum prostate specific antigen and other causes of anxiety in men with metaststic prostate cancer. J Urol 2002;168:2516–2520.

[22] Gramling R, Fiscella K, Xing G, et al. Determinants of patient-oncologist prognostic discordance in advanced cancer. JAMA Oncol 2016;2:1421–1426.

[23] Kessels RPC. Patients' memory for medical information. J R Soc Med 2003;96:219–222.

[24] Rajaram N, Lim ZY, Song CV, et al. Patient-reported outcome measures among breast cancer survivors: a cross-sectional comparison between Malaysia and high-income countries. Psychooncology 2019;28:147–153.

[25] Hansen L, Rosenkranz SJ, Vaccaro GM, et al. Patients with hepatocellular carcinoma near the end of life: a longitudinal qualitative study of their illness experiences. Cancer Nurs 2015;38:E19–E27.

Keywords: hepatocellular carcinoma; systemic treatment; immunotherapy; perception; life expectancy.

Received 11 July 2024; received in revised form 7 August 2024; accepted 8 August 2024; Available online 22 August 2024

Journal of Hepatology, Volume *6*

Supplemental information

Patient and physician expectations regarding disease and treatment of advanced HCC: The prospective PERCEPTION1 study

Jean-Charles Nault, Nanthara Sritharan, Gontran Verset, Ivan Borbath, Marie Lequoy, Manon Allaire, Hélène Regnault, Isabelle Colle, Hans Orlent, Isabelle Sinapi, Christophe Moreno, Edouard Larrey, Sabrina Sidali, Clémence Hollande, Giuliana Amaddeo, Stanislas Pol, Pierre Nahon, Nathalie Ganne-Carrié, Vincent Levy, Coralie Bloch-Queyrat, Paris Liver Cancer Group, Eric Trepo, and Mohammed Bouattour

Patient and physician expectations regarding disease and treatment of advanced HCC: The prospective PERCEPTION1 study

Jean-Charles Nault, Nanthara Sritharan, Gontran Verset, Ivan Borbath, Marie Lequoy, Manon Allaire, Hélène Regnault, Isabelle Colle, Hans Orlent, Isabelle Sinapi, Christophe Moreno, Edouard Larrey, Sabrina Sidali, Clémence Hollande, Giuliana Amaddeo, Stanislas Pol, Pierre Nahon, Nathalie Ganne-Carrié, Vincent Levy, Coralie Bloch-Queyrat, *Paris Liver Cancer Group*, Eric Trepo, Mohammed Bouattour

Table of contents

Fig. S1……………………………………………………………………………..2

Table S1………………………………………………………………………………3

Table S2………………………………………………………………………………4

Table S3………………………………………………………………………………7

Table S4………………………………………………………………………………9

Table S5……………………………………………………………………………10

Table S6……………………………………………………………………………11

Table S7……………………………………………………………………………12

Fig. S1. Flowchart of the study.

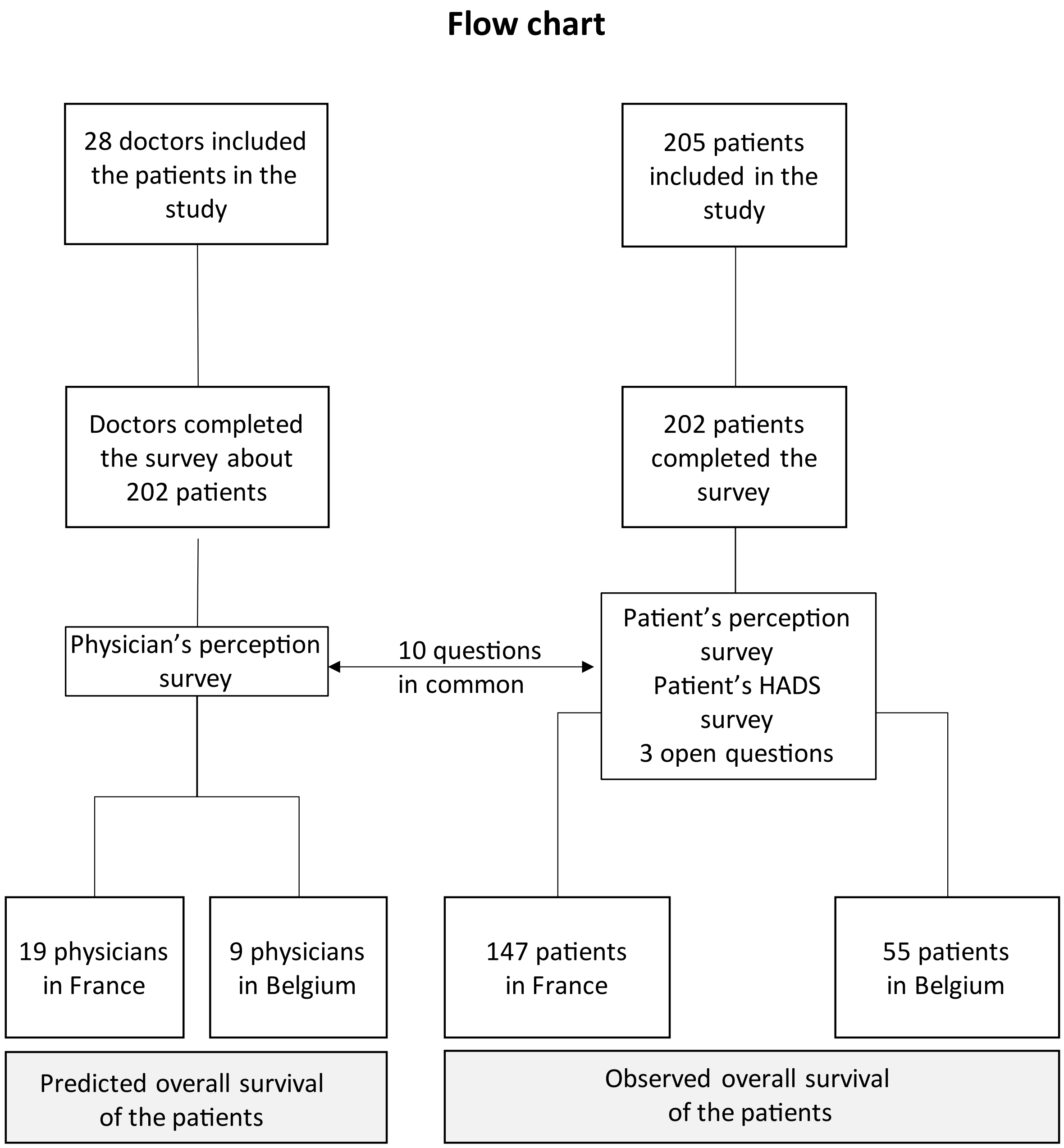

Table S1: Description of the physicians including patients in the PERCEPTION1 study

	Total<br>N= 28 physicians	Belgium<br>N= 9 physicians	France<br>N= 19 physicians
**Gender***			
Male	13 (46.4%)	7 (77.8%)	6 (31.6%)
Female	15 (53.6%)	2 (22.2%)	13 (68.4%)
**Age of the physician (year olds)** $	43.0 [38.0;50.2]	51.0 [43.0;53.0]	41.0 [37.5;48.0]
**Medical Specialty***			
Hepato-gastroenterology	26 (92.9%)	7 (77.8%)	19 (100.0%)
Medical oncology	2 (7.1%)	2 (22.2%)	0 (0%)
**Date of medical graduation***			
Less than 5 years	7 (25.0%)	0 (0%)	7 (36.8%)
Between 5 and 10 years	6 (21.4%)	2 (22.2%)	4 (21.1%)
Between 10 and 20 years	8 (28.6%)	3 (33.3%)	5 (26.3%)
Between 20 and 30 years	6 (21.4%)	4 (44.4%)	2 (10.5%)
More than 30 years	1 (3.6%)	0 (0%)	1 (5.3%)
**Numbers of patients treated for HCC each years***			
Less than 5	0 (0%)	0 (0%)	0 (0%)
Between 5 and 20	9 (32.1%)	7 (77.8%)	2 (10.5%)
Between 20 and 50	5 (17.9%)	0 (0.0%)	5 (26.3%)
Between 50 and 100	6 (21.4%)	1 (11.1%)	5 (26.3%)
More than 100	8 (28.6%)	1 (11.1%)	7 (36.8%)

*numbers (percentage) $ median (interquartile range)

HCC = hepatocellular carcinoma

Table S2: Results of the physician's survey

	Total N= 205 patients	Belgium N= 55 patients	France N= 150 patients	p	Available data
**Survey completed ***	202 (98.5%)	54 (98.2%)	148 (98.7%)	-	205
**Consultation for the announcement of cancer's treatment**					
**1. Length of the consultation - minutes$**	30.0 [25.0;35.0]	30.0 [20.0;35.0]	30.0 [25.0;36.2]	0.06	197
**2. Patient accompanied during the consultation***					
No; alone during the consultation	98 (48.5%)	21 (38.9%)	77 (52.0%)	0.19	202
Yes, by their spouse or another family member	102 (50.5%)	33 (61.1%)	69 (46.6%)		
Yes, by a friend	2 (1.0%)	0 (0%)	2 (1.4%)		
**3. Presence of a dedicated nurse during the consultation***					
During the consultation	46 (22.8%)	16 (29.6%)	30 (20.3%)	0.001	202
After the consultation only	95 (47.0%)	14 (25.9%)	81 (54.7%)		
No consultation nurse	61 (30.2%)	24 (44.4%)	37 (25.0%)		
**4. Do you feel you had enough time to discuss your patient's illness? *%**					
Yes, completely	91 (45.0%)	28 (51.9%)	63 (42.6%)	0.26	202
Yes, somewhat	100 (49.5%)	22 (40.7%)	78 (52.7%)		
No, not really	11 (5.4%)	4 (7.4%)	7 (4.7%)		
No, not at all	0 (0%)	0 (0%)	0 (0%)		
**5. Do you think the patient understood what their treatment will involve based on the explanations you provided and the terms used?* %**					
Yes, completely	56 (27.7%)	18 (33.3%)	38 (25.7%)	0.14	202
Yes, somewhat	129 (63.9%)	29 (53.7%)	100 (67.6%)		
No, not really	17 (8.4%)	7 (13.0%)	10 (6.8%)		
No, not at all	0 (0%)	0 (0%)	0 (0%)		
**6. Do you think you were attentive enough to all the requests the patient wanted to express?* %**					
Yes, completely	64 (31.7%)	27 (50.0%)	37 (25.0%)	0.002	202
Yes, somewhat	132 (65.3%)	25 (46.3%)	107 (72.3%)		
No, not really	6 (3.0%)	2 (3.7%)	4 (2.7%)		
No, not at all	0 (0%)	0 (0%)	0 (0%)		
**7. How did you inform the patient about the possibility of experiencing side effects related to the prescribed treatment?* %**					
In detail	81 (40.5%)	29 (54.7%)	52 (35.4%)	0.03	200
Partially, briefly	113 (56.5%)	24 (45.3%)	89 (60.5%)		
I did not mention it	6 (3.0%)	0 (0%)	6 (4.1%)		
**8. Did you provide an estimation of life expectancy?* %**					
Yes, precisely	13 (6.5%)	5 (9.4%)	8 (5.4%)	0.001	201
Yes, vaguely	87 (43.3%)	33 (62.3%)	54 (36.5%)		
No, not at all	101 (50.2%)	15 (28.3%)	86 (58.1%)		
**9. Do you think the patient trusts your judgment and the treatment you have proposed?* %**					
Yes, completely	50 (25.0%)	24 (45.3%)	26 (17.7%)	<0.001	200

	Total<br>N= 205 patients	Belgium<br>N= 55 patients	France<br>N= 150 patients	p	Available data
Yes, somewhat	141 (70.5%)	27 (50.9%)	114 (77.6%)		
No, not really	4 (2.0%)	0 (0%)	4 (2.7%)		
No, not at all	1 (0.5%)	0 (0%)	1 (0.7%)		
I don't know	4 (2.0%)	2 (3.8%)	2 (1.4%)		
**Understanding of the illness**					
**1. In your opinion, which option best corresponds to your patient's perception of their illness?***					
Accurate perception of the diagnosis	74 (36.8%)	11 (20.8%)	63 (42.6%)	0.01	201
Partially accurate perception	122 (60.7%)	40 (75.5%)	82 (55.4%)		
Inaccurate perception of the diagnosis	5 (2.5%)	2 (3.8%)	3 (2.0%)		
**2. Do you think your patient has realistic expectations regarding the prescribed treatment?***					
Yes	158 (78.6%)	31 (58.5%)	127 (85.8%)	<0.001	201
**3. Do you think the patient's perception of the progression of their illness and overall condition over the next 12 months is realistic? ***					
Yes	119 (61.0%)	27 (50.9%)	92 (64.8%)	0.11	195
**4. In your opinion, what is the life expectancy of the patient? *%**					
more than 5 years	0 (0%)	0 (0%)	0 (0%)	0.04	201
Between 2 à 5 years	43 (21.4%)	16 (30.2%)	27 (18.2%)		
Between 1 à 2 years	109 (54.2%)	30 (56.6%)	79 (53.4%)		
Less than 1 year	49 (24.4%)	7 (13.2%)	42 (28.4%)		
**5. In your opinion, what is the likelihood that your patient will be alive in 2 years? ***					
100%	0 (0%)	0 (0%)	0 (0%)	<0.001	202
75 to 100%	13 (6.4%)	9 (16.7%)	4 (2.7%)		
50 to 75%	43 (21.3%)	17 (31.5%)	26 (17.6%)		
25 to 50%	63 (31.2%)	16 (29.6%)	47 (31.8%)		
0 to 50%	83 (41.1%)	12 (22.2%)	71 (48.0%)		
**Predicted survival of your patient in months** $	17.0 [12.0;21.5]	18.0 [12.0;23.5]	16.0 [10.0;20.0]	0.12	198
**7. In your opinion, what is the probability that the treatment you prescribed will control (at least temporarily) the disease?* %**					
0%	1 (0.5%)	0 (0%)	1 (0.7%)	<0.001	202
Between 0 and 25%	64 (31.7%)	4 (7.4%)	60 (40.5%)		
Between 25 and 50%	64 (31.7%)	17 (31.5%)	47 (31.8%)		
Between 50 and 75%	66 (32.7%)	29 (53.7%)	37 (25.0%)		
Between 75 and 100%	6 (3.0%)	3 (5.6%)	3 (2.0%)		
100%	1 (0.5%)	1 (1.9%)	0 (0%)		
**8. What is the percentage chance that the treatment you prescribed will cause disabling side effects (such as diarrhea, vomiting, significant fatigue, pain...)? *%**					
0%	2 (1.0%)	0 (0%)	2 (1.4%)	0.31	202
Between 0 and 25%	111 (55.0%)	27 (50.0%)	84 (56.8%)		
Between 25 and 50%	73 (36.1%)	20 (37.0%)	53 (35.8%)		
Between 50 and 75%	15 (7.4%)	6 (11.1%)	9 (6.1%)		

	Total N= 205 patients	Belgium N= 55 patients	France N= 150 patients	p	Available data
Between 75 and 100%	1 (0.5%)	1 (1.9%)	0 (0%)		
100%	0 (0%)	0 (0%)	0 (0%)		
**9. Do you believe that for your patient, maintaining quality of life is more important than prolonging life? *[%]**					
Yes, absolutely	55 (27.2%)	10 (18.5%)	45 (30.4%)		
Yes, to some extent	90 (44.6%)	26 (48.1%)	64 (43.2%)		
No, probably not	36 (17.8%)	4 (7.4%)	32 (21.6%)	<0.001	202
No, not at all	7 (3.5%)	2 (3.7%)	5 (3.4%)		
I don't know	14 (6.9%)	12 (22.2%)	2 (1.4%)		

*numbers (percentage) [$] median (interquartile range)

[%] These 10 questions are used to assess the concordance between the patients and the physicians

Table S3: Results of the Hospital Anxiety and Depression scale (HADS) survey

	Total N= 200 patients	Belgium N= 50 patients	France N= 150 patients	Available data
**HADS survey completed***	202 (98.5%)	55 (100.0%)	147 (98.0%)	-
**1. I feel tense or 'wound up***				
Most of the time	19 (9.5%)	5 (9.4%)	14 (9.5%)	200
A lot of the time	27 (13.5%)	11 (20.8%)	16 (10.9%)	
Occasionally	106 (53.0%)	19 (35.8%)	87 (59.2%)	
Not at all	48 (24.0%)	18 (34.0%)	30 (20.4%)	
**2. I still enjoy the things I used to enjoy***				
Definitely as much	97 (48.5%)	28 (52.8%)	69 (46.9%)	200
Not quite so much	76 (38.0%)	22 (41.5%)	54 (36.7%)	
Only a little	18 (9.0%)	2 (3.8%)	16 (10.9%)	
Hardly at all	9 (4.5%)	1 (1.9%)	8 (5.4%)	
**3. I get a sort of frightened feeling as if something awful is about to happen***				
Very definitely & quite badly	37 (18.7%)	9 (17.3%)	28 (19.2%)	198
Yes, but not too badly	36 (18.2%)	10 (19.2%)	26 (17.8%)	
A little, but it doesn't worry me	68 (34.3%)	16 (30.8%)	52 (35.6%)	
Not at all	57 (28.8%)	17 (32.7%)	40 (27.4%)	
**4. I can laugh and see the funny side of things***				
As much as always	108 (54.5%)	30 (56.6%)	78 (53.8%)	198
Not quite so much now	58 (29.3%)	16 (30.2%)	42 (29.0%)	
Definitely not so much now	29 (14.6%)	6 (11.3%)	23 (15.9%)	
Not at all	3 (1.5%)	1 (1.9%)	2 (1.4%)	
**5. Worrying thoughts go through my mind***				
A great deal of the time	30 (15.5%)	14 (28.0%)	16 (11.1%)	194
A lot of the time	43 (22.2%)	7 (14.0%)	36 (25.0%)	
Not too often	58 (29.9%)	13 (26.0%)	45 (31.2%)	
Very little	63 (32.5%)	16 (32.0%)	47 (32.6%)	
**6. I feel cheerful ***				
Never	1 (0.5%)	1 (1.9%)	0 (0.0%)	198
Not often	16 (8.1%)	4 (7.5%)	12 (8.3%)	
Sometimes	32 (16.2%)	7 (13.2%)	25 (17.2%)	
Most of the time	149 (75.3%)	41 (77.4%)	108 (74.5%)	
**7. I can sit at ease and feel relaxed***				
Definitely	95 (48.0%)	27 (50.9%)	68 (46.9%)	198
Usually	75 (37.9%)	20 (37.7%)	55 (37.9%)	
Not often	21 (10.6%)	4 (7.5%)	17 (11.7%)	
Not at all	7 (3.5%)	2 (3.8%)	5 (3.4%)	
**8. I feel as if I am slowed down***				
Nearly all the time	18 (9.1%)	5 (9.4%)	13 (9.0%)	197
Very often	50 (25.4%)	11 (20.8%)	39 (27.1%)	
Sometimes	78 (39.6%)	17 (32.1%)	61 (42.4%)	
Not at all	51 (25.9%)	20 (37.7%)	31 (21.5%)	

	Total N= 200 patients	Belgium N= 50 patients	France N= 150 patients	Available data
**9. I get a sort of frightened feeling like 'butterflies' in the stomach***				
Not at all	91 (46.4%)	24 (45.3%)	67 (46.9%)	196
Occasionally	88 (44.9%)	26 (49.1%)	62 (43.4%)	
Quite often	11 (5.6%)	2 (3.8%)	9 (6.3%)	
Very often	6 (3.1%)	1 (1.9%)	5 (3.5%)	
**10. I have lost interest in my appearance***				
Definitely	17 (8.6%)	7 (13.2%)	10 (6.9%)	197
I don't take as much care as I should	28 (14.2%)	9 (17.0%)	19 (13.2%)	
I may not take as much care	52 (26.4%)	7 (13.2%)	45 (31.2%)	
I take just as much care as ever	100 (50.8%)	30 (56.6%)	70 (48.6%)	
**11. I feel restless as if I have to be on the move**				
Very much indeed	21 (10.7%)	6 (11.3%)	15 (10.5%)	196
Quite a lot	33 (16.8%)	11 (20.8%)	22 (15.4%)	
Not very much	75 (38.3%)	15 (28.3%)	60 (42.0%)	
Not at all	67 (34.2%)	21 (39.6%)	46 (32.2%)	
**12. I look forward to enjoyment to things***				
As much as I ever did	76 (39.2%)	20 (40.0%)	56 (38.9%)	194
Rather less than I used to	79 (40.7%)	21 (42.0%)	58 (40.3%)	
Definitely less than I used to	28 (14.4%)	7 (14.0%)	21 (14.6%)	
Hardly at all	11 (5.7%)	2 (4.0%)	9 (6.2%)	
**13. I get sudden feelings of panic***				
Very often indeed	1 (0.5%)	1 (1.9%)	0 (0.0%)	198
Quite often	20 (10.1%)	5 (9.6%)	15 (10.3%)	
Not very often	62 (31.3%)	16 (30.8%)	46 (31.5%)	
Not at all	115 (58.1%)	30 (57.7%)	85 (58.2%)	
**14. I can enjoy a good book or radio or TV program***				
Often	137 (69.2%)	37 (71.2%)	100 (68.5%)	198
Sometimes	44 (22.2%)	10 (19.2%)	34 (23.3%)	
Not often	8 (4.0%)	3 (5.8%)	5 (3.4%)	
Very seldom	9 (4.5%)	2 (3.8%)	7 (4.8%)	
**HADS score (anxiety)** $^{\$}$	6.0 [4.0;9.0]	6.0 [3.0;10.0]	6.0 [4.0;9.0]	188
**HADS score (depression)** $^{\$}$	4.0 [2.0;7.2]	4.0 [1.2;7.0]	5.0 [3.0;7.8]	192
**HADS score (total)** $^{\$}$	11.0 [7.0;16.0]	10.5 [5.2;16.8]	11.0 [7.0;15.0]	185
**HADS score by category***				
No symptoms (0 to 7)	58 (31.4%)	16 (34.8%)	42 (30.2%)	185
Doubtful symptoms (8 to 10)	30 (16.2%)	7 (15.2%)	23 (16.5%)	
Certain symptoms (11 to more)	97 (52.4%)	23 (50.0%)	74 (53.2%)	

*numbers (percentage) $^{\$}$ median (interquartile range), HADS = Hospital Anxiety and Depression Scale

Table S4: Association between patient's factors and HADS using multivariate linear regression

	**β ± SE**	**p**
Country – France (ref : Belgium)	0.24 ± 1.01	0.81
Non tumor fibrosis F2/F3 (ref : F0/F1)	2.26 ± 1.83	0.22
Non tumor fibrosis F4	1.70 ± 1.17	0.15
Serum AFP level (logarithmic)	0.34 ± 0.15	**0.03**
Performance status 1/2 (ref : 0)	1.34 ± 0.94	0.15
Unemployed (Job seeker) (ref: active worker)	2.76 ± 2.18	0.21
Unable to work, disabled	3.63 ± 1.81	**0.04**
Retired	1.08 ± 1.28	0.40

AFP= alpha foetoprotein, Ref = reference, SE= standard error of coefficients

Table S5: Multivariate linear mixed-effects model of baseline factors associated with predicted death.

	β ± SE	p
Gender of the patient (Female) (ref : Male)	3.57 ± 1.43	**0.02**
Age of the patient (years old)	0.07 ± 0.05	0.14
Non tumor fibrosis F2/F3 (ref : F0/F1)	0.64 ± 2.08	0.76
Non tumor fibrosis F4	-1.37 ± 1.36	0.31
BCLC C (ref : BCLC B)	-2.48 ± 1.21	**0.04**
Child Pugh score – B (ref : A)	-1.87 ± 1.30	0.15
Serum AFP level (logarithmic)	-0.39 ± 0.15	**0.01**
Performance status – 1/2 (ref : 0)	-2.95 ± 1.04	**0.005**
Previous treatment for HCC – Yes (ref : No)	1.11 ± 1.06	0.29
Numbers of patients treated for HCC each year by the doctor between 50 and 100 (ref ≤ 50)	2.97 ± 2.01	0.15
Numbers of patients treated for HCC each year by the doctor > 100	1.49 ± 2.14	0.49

AFP= alpha foetoprotein, BCLC = Barcelona clinic cancer, HCC = hepatocellular carcinoma, Ref = reference, SE= standard error of coefficients

Table S6: Baseline factors associated with observed death using Cox proportional hazards model

	**HR [95%CI]**	**p**
Country – France (ref : Belgium)	0.80 [0.50-1.30]	0.37
BCLC C (ref : BCLC B)	1.91 [1.16-3.13]	**0.01**
Child Pugh score – B (ref : A)	1.86 [1.15-3.01]	**0.01**
Serum AFP level (logarithmic)	1.03 [0.97-1.09]	0.36
Performance status – 1/2 (ref : 0)	1.67 [1.12-2.47]	**0.01**
Type of treatment – Sorafenib	22.77 [5.08-102.12]	**< 0.001**
HADS score total	1.02 [0.99-1.05]	0.26
Married, in a relationship (ref: Single)	0.65 [0.38-1.13]	0.13
Divorced, separated	1.40 [0.68-2.89]	0.36
Widowed	0.91 [0.42-1.96]	0.81

AFP= alpha foetoprotein, BCLC = Barcelona clinic cancer, HADS = Hospital Anxiety and Depression Scale, HR= Hazard Ratio, 95% CI = 95% confidence interval

Table S7: correlation between results of HADS survey with the answers to the patient's survey using multivariate linear regression

	Total<br>N= 205 patients	β ± SE	P value
**Socio-cultural level and lifestyle**			
**1. What is your marital/family status?**			
Single	26 (13.0%)	reference	reference
Married, in a relationship	130 (65.0%)	-0.127 ± 1.363	> 0,9
Divorced, separated	23 (11.5%)	0.785 ± 1.832	0.67
Widowed	21 (10.5%)	1.493 ± 1.857	0.42
**2. At home, do you live?**			
Alone	47 (23.5%)	reference	reference
With someone constantly	141 (70.5%)	-1.029 ± 1.081	0.34
With someone intermittently present	12 (6.0%)	-1.666 ± 2.101	0.43
**3. Regarding your education, what is the highest level of your degree?**			
Primary school	47 (24.0%)		
High school : general education	52 (26.5%)	1.253 ± 1.309	0.34
High school : vocational education	61 (31.1%)	0.347 ± 1.224	0.78
University or higher education	36 (18.4%)	-0.955 ± 1.392	0.49
**4. What is your current employment status?**			
Active worker	32 (16.0%)		
Unemployed (Job seeker)	11 (5.5%)	2.708 ± 2.218	0.22
Unable to work, disabled	20 (10.0%)	3.676 ± 1.841	0.047
Retired	137 (68.5%)	0.795 ± 1.285	0.54
**5. What is your current alcohol consumption?**			
Every day/every week	29 (14.5%)		
Occasional (less than once per week)	39 (19.5%)	0.43 ± 1.575	0.78
Never	132 (66.0%)	-0.094 ± 1.332	> 0,9
**Consultation for the announcement of cancer's treatment**			
**1. Did you have enough time to discuss your illness with your doctor? * [%]**			
Yes, completely	150 (74.6%)	reference	reference
Mostly yes	44 (21.9%)	2.258 ± 1.077	0.04
Mostly no + no, not at all	7 (3.5%)	3.344 ± 2.34	0.15
**2. Did the doctor explain the type of treatment you will receive in a way that was understandable to you? * [%]**			
Yes, completely	172 (85.6%)	reference	reference
Mostly yes	25 (12.4%)	2.229 ± 1.327	0.09
Mostly no + no, not at all	4 (2.0%)	5.734 ± 2.97	0.05
**3. Did the doctor listen to what you had to say? * [%]**			
Yes, completely	177 (88.1%)	reference	reference
Mostly yes	23 (11.4%)	1.633 ± 1.377	0.24
Mostly no + no, not at all	1 (0.5%)	0.875 ± 5.774	0.88
**4. Did the doctor discuss the possibility of treatment-related side effects with you? * [%]**			
Yes, in detail	161 (80.5%)	reference	reference
Yes, briefly	29 (14.5%)	0.561 ± 1.25	0.65
No, not at all	10 (5.0%)	3.992 ± 1.9	0.04
**5. Did the doctor discuss your life expectancy with you? * [%]**			
Yes, in detail	37 (18.7%)	reference	reference
Yes, briefly	43 (21.7%)	1.201 ± 1.436	0.40
No, not at all	118 (59.6%)	-0.409 ± 1.225	0.74
**6. Do you wish to have specific numerical information (in terms of months or percentage) about your life expectancy?**			
Yes	78 (39.0%)	reference	reference
No	80 (40.0%)	-3.654 ± 0.99	< 0,001
I don't know	42 (21.0%)	-2.598 ± 1.168	0.03
**7. Were you involved as much as you wanted in the choice of the proposed treatment?***			
Yes, completely	104 (52.0%)	reference	reference
Mostly yes	74 (37.0%)	0.502 ± 0.954	0.60
Mostly no	15 (7.5%)	3.077 ± 1.731	0.08

No, not at all	6 (3.0%)	2.519 ± 2.467	0.31
I did not wish to be involved	1 (0.5%)	4.595 ± 5.675	0.42
**8. Overall, how do you evaluate the quality of your consultation?***			
Excellent	108 (54.3%)	reference	reference
Very good	81 (40.7%)	1.445 ± 0.937	0.12
Average	9 (4.5%)	2.313 ± 1.939	0.23
Poor + very poor	1 (0.5%)	-1.572 ± 5.657	0,78
**9. Do you trust your doctor's judgment regarding the proposed treatment?* #**			
Yes, completely	171 (85.5%)	reference	reference
Mostly yes	28 (14.0%)	4.151 ± 1.232	0.0009
Mostly no + no, not at all	1 (0.5%)	0.487 ± 5.392	> 0,9
**Understanding of the disease**			
**1. How do you assess your current health status?**			
I feel healthy despite my illness, and I hope to recover	125 (62.8%)	reference	reference
I feel healthy despite my illness, but I won't be able to recover	43 (21.6%)	3.352 ± 1.033	0.001
I feel very sick due to my illness, but I hope to recover	21 (10.6%)	6.362 ± 1.407	< 0,001
I feel very sick due to my illness, and I won't be able to recover	6 (3.0%)	9.247 ± 2.409	0.0002
I have no opinion	4 (2.0%)	6.353 ± 2.944	0.03
**2. In your opinion, what is the stage of your cancer?**			
I do not have cancer	2 (1.0%)	reference	reference
Early, very localized cancer	27 (13.5%)	0.319 ± 4.613	> 0,9
Intermediate stage cancer	64 (32.0%)	1.742 ± 4.519	0.70
Advanced stage cancer	56 (28.0%)	2.819 ± 4.528	0.53
I don't know	51 (25.5%)	2.313 ± 4.544	0.61
**3. In your opinion, what could be the impact of the illness in terms of life expectancy? %**			
I have a life expectancy of more than 5 years	100 (63.3%)	reference	reference
I have a life expectancy of 2 to 5 years	37 (23.4%)	2.04 ± 1.149	0.08
I have a life expectancy of less than 2 years	21 (13.3%)	3.14 ± 1.354	0.02
**Understanding of the treatment**			
**1. Among the following options, which one best corresponds to what your doctor told you during the consultation regarding your treatment– N(%)**			
My cancer will be cured	15 (7.5%)	reference	reference
My cancer can be cured if the treatment works	89 (44.5%)	3.683 ± 1.739	0.04
My cancer cannot be cured, but we will try to control the disease with treatment	70 (35.0%)	5.397 ± 1.779	0.003
I don't know	15 (7.5%)	6.453 ± 2.006	0.002
**2. What percentage chance do you believe there is that your treatment will shrink or stop the progression of your cancer? %**			
Between 0 and 25%	13 (7.3%)	reference	reference
Between 25 and 50%	41 (23.0%)	1.64 ± 2.241	0.47
Between 50 and 75%	50 (28.1%)	0.503 ± 1.994	0.80
Between 75 and 100%	43 (24.2%)	-0.748 ± 2.072	0.72
100%	31 (17.4%)	-2.217 ± 2.22	0,32
**3. Is maintaining your quality of life more important to you than living longer?– N(%)**			
Yes, completely	73 (37.2%)	reference	reference
Mostly yes	81 (41.3%)	1.725 ± 1.009	0.09
Mostly no	31 (15.8%)	0.225 ± 1.351	0.87
No, not at all	11 (5.6%)	-2.175 ± 2.035	0.29
**4. What percentage chance do you believe there is that the treatment will cause disabling side effects (such as diarrhea, vomiting, significant fatigue, pain...)?– N(%)%**			
0%	16 (8.7%)	reference	reference
Between 0 and 25%	64 (35.0%)	1.856 ± 1.719	0.28
Between 25 and 50%	71 (38.8%)	4.354 ± 1.687	0.01
Between 50 and 75%	28 (15.3%)	3.654 ± 1.928	0.06
Between 75 and 100%	4 (2.1%)	7.591 ± 3.574	0.04
**5. From how many months of life expectancy gain are you willing to accept significant side effects (such as nausea/vomiting, significant fatigue, or pain)?– N(%)%**			
0 to 3 months	37 (22.4%)	reference	reference
3 to 6 months	21 (12.7%)	1.335 ± 1.669	0.43
6 to 12 months	28 (17.0%)	0.006 ± 1.611	> 0,9
12 to 24 months	25 (15.2%)	0.401 ± 1.648	0.81
> 24 months	54 (32.7%)	0.267 ± 1.29	0.84

**6. I would like to try treatments for my cancer if they can make me live longer, even if it is very likely that they:**			
**6.1- Present a high level of side effects (such as nausea/vomiting, significant fatigue, or pain)– N(%)**			
Completely agree	47 (23.9%)	reference	reference
Agree	76 (38.6%)	1.379 ± 1.154	0.23
Disagree	27 (13.7%)	2.373 ± 1.508	0.12
Strongly disagree	7 (3.6%)	0.685 ± 2.532	0.79
No opinion	40 (20.3%)	-1.509 ± 1.323	0.26
**6.2- Require me to be bedridden and render me unable to use the bathroom or toilet without assistance – N(%)**			
Completely agree	15 (7.8%)	reference	reference
Agree	27 (14.0%)	3.469 ± 2.018	0.09
Disagree	59 (30.6%)	2.579 ± 1.775	0.15
Strongly disagree	54 (28.0%)	4.555 ± 1.842	0.01
No opinion	38 (19.7%)	1.962 ± 1.895	0.30
**6.3- Require me to rely on help from my family and friends to carry out daily activities (such as shopping and managing money)– N(%)**			
Completely agree	29 (15.0%)	reference	reference
Agree	60 (31.1%)	1.22 ± 1.402	0.39
Disagree	39 (20.2%)	-0.468 ± 1.494	0.75
Strongly disagree	36 (18.7%)	2.758 ± 1.606	0.09
No opinion	29 (15.0%)	-1.137 ± 1.646	0.49
**6.4- Impair my memory or concentration – N(%)**			
Completely agree	15 (7.9%)	reference	reference
Agree	44 (23.0%)	2.941 ± 1.806	0.11
Disagree	56 (29.3%)	2.515 ± 1.768	0.16
Strongly disagree	33 (17.3%)	4.632 ± 1.892	0.02
No opinion	43 (22.5%)	1.233 ± 1.847	0.51
**6.5- Make me occasionally confused and disoriented – N(%)**			
Completely agree	16 (8.6%)	reference	reference
Agree	40 (21.4%)	4.042 ± 1.823	0.03
Disagree	51 (27.3%)	2.089 ± 1.763	0.24
Strongly disagree	34 (18.2%)	3.204 ± 1.902	0.09
No opinion	46 (24.6%)	1.515 ± 1.802	0.40

We regroup some categories of answers for the analysis when the numbers
